# Immunogenicity and protective efficacy of a pan-fungal vaccine in preclinical models of aspergillosis, candidiasis, and pneumocystosis

**DOI:** 10.1093/pnasnexus/pgac248

**Published:** 2022-11-04

**Authors:** Emily Rayens, Whitney Rabacal, Hubertine M E Willems, Gabrielle M Kirton, James P Barber, Jarrod J Mousa, Brandi N Celia-Sanchez, Michelle Momany, Karen A Norris

**Affiliations:** Center for Vaccines and Immunology, University of Georgia, Athens, GA 30602, USA; Center for Vaccines and Immunology, University of Georgia, Athens, GA 30602, USA; Center for Vaccines and Immunology, University of Georgia, Athens, GA 30602, USA; Center for Vaccines and Immunology, University of Georgia, Athens, GA 30602, USA; Department of Infectious Diseases, University of Georgia, Athens, GA 30602, USA; Center for Vaccines and Immunology, University of Georgia, Athens, GA 30602, USA; Fungal Biology Group, Department of Plant Biology, University of Georgia, Athens, GA 30602, USA; Fungal Biology Group, Department of Plant Biology, University of Georgia, Athens, GA 30602, USA; Center for Vaccines and Immunology, University of Georgia, Athens, GA 30602, USA

**Keywords:** mycoses, *Aspergillus*, *Pneumocystis*, *Candida*, fungal vaccine

## Abstract

Invasive fungal infections cause over 1.5 million deaths worldwide. Despite increases in fungal infections as well as the numbers of individuals at risk, there are no clinically approved fungal vaccines. We produced a “pan-fungal” peptide, NXT-2, based on a previously identified vaccine candidate and homologous sequences from *Pneumocystis, Aspergillus,Candida*, and *Cryptococcus*. We evaluated the immunogenicity and protective capacity of NXT-2 in murine and nonhuman primate models of invasive aspergillosis, systemic candidiasis, and pneumocystosis. NXT-2 was highly immunogenic and immunized animals had decreased mortality and morbidity compared to nonvaccinated animals following induction of immunosuppression and challenge with *Aspergillus, Candida*, or *Pneumocystis*. Data in multiple animal models support the concept that immunization with a pan-fungal vaccine prior to immunosuppression induces broad, cross-protective antifungal immunity in at-risk individuals.

Significance Statement Despite the substantial impact of invasive fungal infections (IFIs) in an expanding immunocompromised patient population, there are no clinically approved fungal vaccines and alternative prophylaxis strategies are limited. The present work demonstrates the efficacy of a recombinant peptide vaccine in developing protective antibodies against *Aspergillus, Candida*, and *Pneumocystis* challenges in preclinical animal models. This demonstrably pan-fungal vaccine may have the potential to reduce the morbidity and mortality associated with the three most prevalent causes of IFIs.

## Introduction

Invasive fungal infections (IFIs) are a major public health concern with high morbidity and mortality rates, even with medical intervention (1). In hospitalized patients, fungal diagnosis is associated with doubled length of stay, cost of stay, and risk of mortality compared to patients without fungal infections (2). IFIs generally occur in conjunction with other health issues that are a consequence of mild to severe immunocompromised states. Individuals at highest risk are those with immunosuppression due to stem cell or solid organ transplant antirejection treatments (3, 4), cancer patients (5), individuals undergoing treatment for inflammatory diseases (6–8), and those with uncontrolled or undiagnosed HIV (9). Individuals with other conditions including diabetes, chronic lung disease such as asthma and emphysema, and congenital or acquired immunosuppressive states are also at increased risk of IFIs (10). Additionally, IFIs contribute to increased morbidity and mortality in individuals with other respiratory infections, including COVID-19, influenza, and tuberculosis (11–16). The majority of IFIs are caused by fungal organisms of three genera: *Aspergillus, Candida*, and *Pneumocystis* (17). Invasive presentations of these pathogens affect roughly 40% of the estimated 13.5 million people impacted by fungal infections worldwide. These same three IFIs are responsible for about 55% of the 1.6 million fungal disease deaths reported globally (1). Including asthmatic and chronic presentations, these three pathogens are estimated to be responsible for over 90% of fungal deaths every year (18).

Current drug therapies and prophylactic regimens for IFIs include azoles and are generally effective. However, drug–drug interactions, low or variable environmental exposure, daily intravenous administration, and increasing antifungal drug resistance (19–23) all limit currently available therapeutic strategies. Suboptimal therapeutic options and delayed diagnoses contribute to mortality rates between 20% and 70% (24, 25). Despite a rise in the number of at-risk individuals and associated fungal infections (17), there are no clinically approved antifungal vaccines. Further, many of these patients are at risk for multiple pathogenic fungi, complicating existing prophylactic strategies as there is not currently a single option that is effective and cross-protective in this population (26).

Our previous work has investigated the host immune response to opportunistic fungal pathogens, including *Pneumocystis jirovecii* (previously *Pneumocystis carinii*), where we identified PC.KEX1 (previously KEX1) as a vaccine candidate, with amino acid sequence homology to a conserved region of the *Pneumocystis* endoprotease Kexin (27–29). We have demonstrated that immunization of nonhuman primates (NHPs) with recombinant PC.KEX1 was protective against pneumocystosis in a model of HIV and *Pneumocystis* coinfection (30). We further showed that antibody levels to *Pneumocystis* KEX1, due to natural exposure to the ubiquitous organism, correlated with decreased frequency of *Pneumocystis* pneumonia in HIV-infected individuals and in simian immunodeficiency virus (SIV)-infected NHPs (31, 32). The *Pneumocystis* PC.KEX1 sequence is highly conserved among pathogenic fungi, including *Aspergillus fumigatus* (33). To test the extent of protection of the KEX1 peptide against other fungal pathogens, we generated an *Aspergillus*-specific KEX1 recombinant homolog, AF.KEX1 (33). In murine models of combination drug-induced immunosuppression, mice vaccinated with AF.KEX1 and challenged with a lethal dose of *A. fumigatus* following immunosuppression had decreased rates of mortality and lower lung organism burden compared to sham-vaccinated controls. The lung fungal burden was inversely correlated with the peak anti-AF.KEX1 IgG titer achieved following vaccination, supporting a role for anti-KEX1 antibodies in protection.

Based on the evidence of protective efficacy of the KEX1-based vaccine candidates against *Pneumocystis* and *Aspergillus*, we designed a “pan-fungal” protein based on a consensus amino acid sequence of the conserved KEX1 regions of multiple fungal pathogens. In this study, we evaluate the use of this recombinant pan-fungal protein (NXT-2) in preventing mortality and ameliorating morbidity related to fungal disease challenge in immunosuppressed models of aspergillosis, candidiasis, and pneumocystosis.

## Materials and Methods

### Animals

All studies were approved by the Institutional Animal Care and Use Committee of the University of Georgia. Adult, Chinese-origin rhesus macaques (*Macaca mulatta*) were purchased from vendors approved by the University of Georgia. 6–8-week-old CD-1 and BALB/c mice were purchased from Charles River Laboratories. An overview of all animal studies can be found in Table S1.

### Vaccine construction and purification

**Fig. 1.**
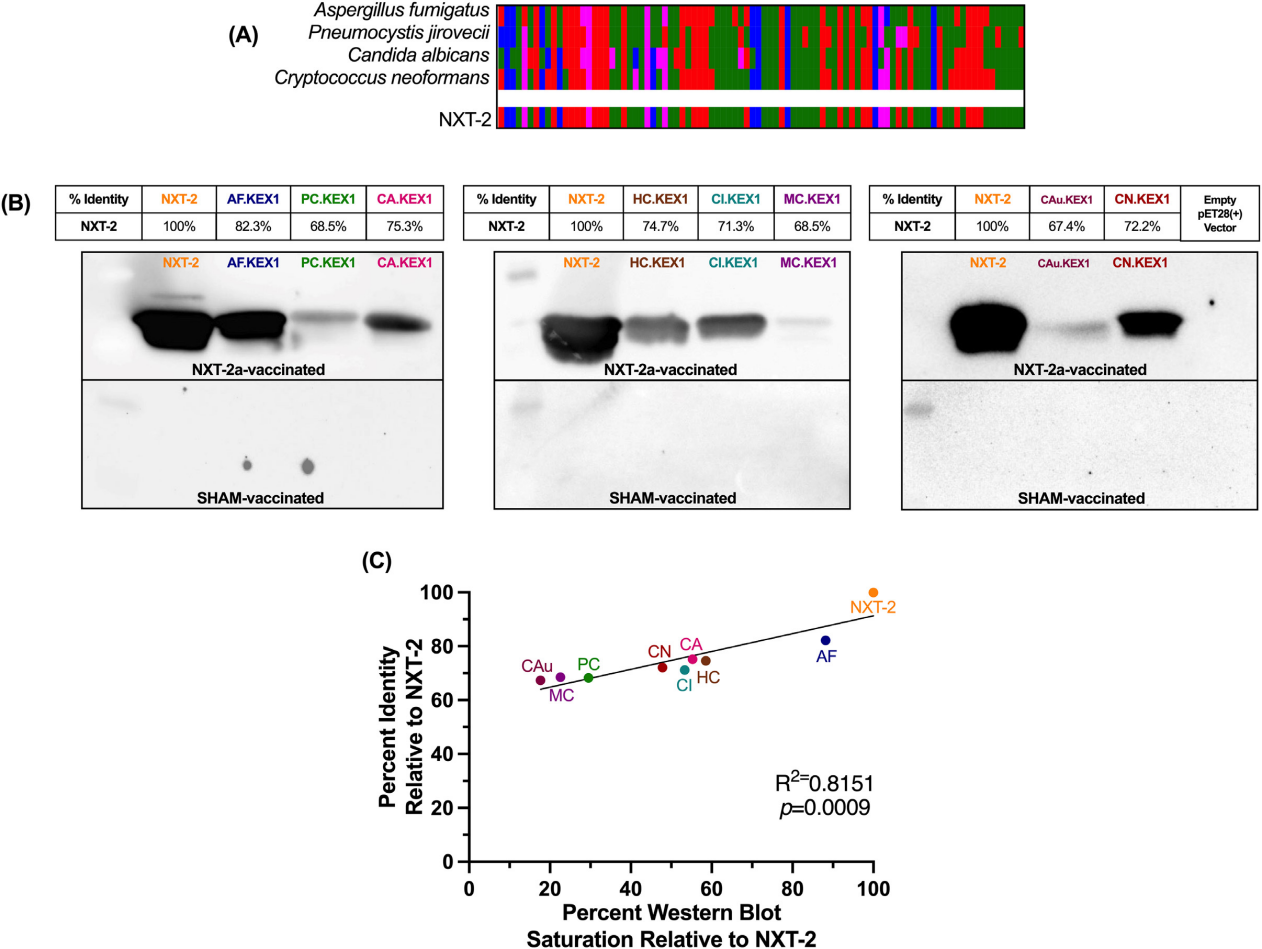
NXT-2 immunization generates antifungal cross-reactive antibodies. (A) The NXT-2 sequence was generated to a consensus sequence with homology to KEX1 regions of *Pneumocystis* (PC.KEX1),*Aspergillus* (AF.KEX1),*Candida* (CA.KEX1), and *Cryptococcus* (CN.KEX1), by amino acid type (Clustal Omega). (B) Percent identity of each KEX1 variant against NXT-2 and the western blots developed using plasma from NHPs immunized with NXT-2a + alum or PBS + alum. Antibodies to NXT-2a bind to NXT-2 (Blot 1, lane 2), AF.KEX1 (Blot 1, lane 3), PC.KEX1 (Blot 1, lane 4), and CA.KEX1 (Blot 1, lane 5). Antibodies to NXT-2a further bind to *Histoplasma capsulatum* KEX1 (HC.KEX2; Blot 2, lane 3), *Coccidioides immitis* KEX1 (CI.KEX1; Blot 1, lane 4), *Mucor circinelloides* KEX1 (MC.KEX1; Blot 1, lane 5), *Candida auris* KEX1 (CAu.KEX1; Blot 1, lane 3), and *C. neoformans* KEX1 (CN.KEX1; Blot 1, lane 4). (C) The correlation of % identity and the % western blot saturation quantified by ImageJ relative to NXT-2 (*P* = 0.0009).

To create a pan-fungal consensus sequence of KEX1, multisequence alignments of KEX1 peptide sequences from *P. jirovecii* (accession number ACB98639.1, residue number 87 to 174), *A. fumigatus* (accession number XM746441, residue number 300 to 389), *Candida albicans* (accession number AF022372, residue number 314 to 402), and *Cryptococcus neoformans* (accession number XP572303, residue number 291 to 380) were performed using Clustal Omega (http://www.ebi.ac.uk/Tools/msa/clustalo/) to analyze sequence identity and similarity (Fig. 1A). As shown in Fig. 1(B), the resulting 90-mer pan-fungal sequence (NXT-2; 5′-PDDGKTMEGPDILVLRAFINGVQNGRDGKGSIYVFASGNGGGFEDNCNFDGYTNSIYSITVGAIDRKGLHPSYSEACSAQLVVTYSSGSG-3′) has approximately 82%, 75%, and 69% amino acid sequence identities, respectively, with the amino acid sequences of *A. fumigatus* KEX1 (AF.KEX1 (33)), *C. albicans* KEX1 (CA.KEX1), and *Pneumocystis* KEX1 (PC.KEX1; previously KEX1 (30)). A 6xHis-tag and associated linker was added to the NXT-2 sequence for purification by affinity chromatography and two amino acids (methionine and glycine) were added to keep the sequence in frame. The expanded sequence (5′-MGPDDGKTMEGPDILVLRAFINGVQNGRDGKGSIYVFASGNGGGFEDNCNFDGYTNSIYSITVGAIDRKGLHPSYSEACSAQLVVTYSSGSGRDPNSSSVDKLAAALEHHHHHH-3′) was cloned with restriction sites NcoI and BamHI into the pET28b(+) expression vector (Novagen) in *Escherichia coli* BL21(DE3) pLysS (ThermoFisher Scientific). AF.KEX1, CA.KEX1, and PC.KEX1 proteins were constructed as described here and purified as previously described (30, 33).

#### Western blot analysis

Western blot analysis was used to assess the cross-reactivity of vaccine-generated anti-NXT-2 antibodies to proteins that have been previously established as protective vaccine candidates (30, 33). In order to minimize any cross-reactivity attributable to the linker or histidine tags utilized in the KEX1 recombinant constructs, NHPs were vaccinated with a modified NXT-2 recombinant protein lacking any vector-associated amino acids (NXT-2a) and aluminum hydroxide. Purified recombinant NXT-2, AF.KEX1, PC.KEX1, and CA.KEX1 proteins were run on SDS-PAGE gels, transferred to a nitrocellulose membrane, blocked in 5% nonfat milk, and incubated with plasma obtained from NXT-2a-immunized NHPs. Blots were washed, incubated with horseradish peroxidase-conjugated IgG secondary antibody, and developed according to standard protocols. To evaluate further cross-reactivity of anti-NXT-2 antibodies, the same cloning, purification, and western blot processes was repeated with recombinant 90-mer KEX1 proteins from *Histoplasma capsulatum* (HC.KEX1; accession number EER45430.1, residue number 316 to 402), *Mucor circinelloides* (MC.KEX1; accession nnumber EPB86942.1, residue number 290 to 378), *Coccidioides immitis* (CI.KEX1; XP_572303.1, residue number 319 to 405), *Candida auris* (CAu.KEX1; accession number NW_017263955.1, residue number 304 to 393), and *C. neoformans* (CN.KEX1). Western blot intensity was quantified by ImageJ according to NIH guidelines and presented relative to the intensity of anti-NXT-2a plasma on an NXT-2 band (34).

### Anti-NXT-2 antibody functionality: surface binding of *Aspergillus* and *Candida*

*Aspergillus fumigatus* AF293 conidia were grown and harvested as previously described (33). The cultured *A. fumigatus* conidia were then allowed to swell in RPMI at 37°C for 5 hours (35). *Candida albicans* cells were collected via vaginal lavage of mice intravaginally challenged with *C. albicans* three days prior, filtered, and washed three times with 1x PBS. All cells were then fixed in 4% paraformaldehyde at 4°C for 1 hour in a 96-well plate with 2 × 10^6^ cells per well. After blocking the cells with 5% fetal bovine serum solution (36) for 30 minutes at room temperature, the cells were washed three times with 1x PBS and resuspended in 100 μL of anti-NXT-2a or antialum mouse plasma diluted at 1:100 in 1x PBS. After incubating in plasma for an hour at room temperature, the cells were washed as above and 100 μL combined of Rhodamine Red antimouse IgG secondary antibody (ThermoFisher, 10 μg/mL) and BV510 CD45 (Biolegend, 0.5 μg/mL) were added. After 1 hour of incubation at room temperature, the cells were resuspended in 200 μL of PBS.

A volume of 20 μL of stained cells were added to glass slides and coverslips were secured with a drop of Permount mounting medium (Fisher Scientific). The images were taken using a Nikon Ti Eclipse/A1R laser scanning confocal microscope (Nikon Instruments Inc.). The remaining cell suspension, up to 200,000 events, was acquired using NovoCyte Quanteon flow cytometer (Agilent). *Candida albicans* were identified in flow analysis of the lavage sample by size and as CD45-. Flow cytometry data were analyzed using FlowJo software (BD Biosciences).

### Immunization of mice

12 BALB/c and 15 CD-1 mice were immunized subcutaneously at the base of the tail with 45 μg NXT-2, AF.KEX1, or CA.KEX1 prepared 1:1 with the water: squalene adjuvant TiterMax (Sigma–Aldrich, Inc.) according to the manufacturer’s guidelines. An additional 13 BALB/c and 15 CD-1 were sham immunized with PBS + TiterMax. Blood was collected at day of immunization, and at 14 and 28 days following. Plasma samples were stored at –80°C until analyzed. Additional details are provided in Table S1.

### ELISA assay

Microtiter plates (Immunolon 4HBX; Thermo Fisher Scientific) were coated with purified NXT-2 at 5 µg/mL in PBS. Heat-inactivated plasma samples were diluted 1:100 in blocking buffer (PBS with 5% nonfat milk) and 1:2 serial dilutions were made to determine endpoint titers, as previously described (30, 33). Goat antimouse and goat antimonkey immunoglobulin conjugated horseradish peroxidase (Southern Biotech, Accurate Chemical) were used for detection, and plates were developed with TMB (Fisher Scientific). Naive (uninfected, NXT-2-negative by antibody titer) mouse and NHP plasma samples were used as negative controls.

### Immunosuppression and *A. fumigatus* challenge, monitoring, and fungal burden

A total of 28 days following immunization, BALB/c mice were subcutaneously injected with 125 mg/kg hydrocortisone (Sigma–Aldrich, Inc.) every three days and 1 mg/kg tacrolimus (PKC Pharmaceuticals) intraperitoneally daily, as described (33, 37). This regimen began three days prior to challenge and continued for the duration of the study. Trimethoprim sulfamethoxazole (TMP/SMX; Med-Vet International) was added to the drinking water to prevent opportunistic bacterial infections during immunosuppressive drug dosing.

*Aspergillus fumigatus* AF293 conidia were grown as previously described (33). Mice were challenged with 5 × 10^6^ conidia in 40 μL PBS via intranasal inoculation. Following challenge, mice were monitored twice daily for changes in weight and temperature. If weight loss greater than 20% or body temperature below 29°C was recorded, the animals were euthanized. At 10 days following challenge, all remaining animals were euthanized, and lungs were collected for analysis. At study termination, right lungs were preserved in 10% neutral buffer formalin, and subsequently the fixed lung tissue was embedded in paraffin, cut, and stained with Grocott’s modified methenamine silver (GMS) stain. Images of five distinct fields were taken and fungal burden quantified as previously described (33, 38). Percent weight loss was calculated from the baseline body weight measured at time of challenge.

### Immunosuppression and *C. albicans* challenge, monitoring, and fungal burden

A total of 28 days following immunization, CD-1 mice were administered cyclophosphamide-based regimens as previously described in models of invasive candidiasis (36, 39) with 200 mg/kg intraperitoneal cyclophosphamide (Sigma–Aldrich, Inc.) and 250 mg/kg subcutaneous cortisone acetate (Sigma–Aldrich, Inc.) injections 2 days prior and 3 days following challenge. Neutropenia as a result of this regimen was confirmed in a pilot cohort by depletion in neutrophil count in Wright-stained blood smears (40) prepared prior to and three days following immunosuppression (Figure S1). *Candida albicans* SC5314 cells were cultured in Yeast Extract Peptone Dextrose (YPD) broth overnight at 30°C, shaking at 225 rpm. Following growth*, C. albicans* cells were washed in phosphate-buffered saline (PBS) by centrifugation and counted on a hemocytometer. Mice were inoculated with 5 × 10^5^ colony forming units (CFUs) in 100 μL PBS via intravenous tail inoculation. Following challenge, mice were monitored twice daily for changes in weight, temperature, and physical condition. If weight loss was greater than 20% or body temperature was below 29°C, or mice became moribund, the animals were euthanized. At 10 days following challenge, all remaining animals were euthanized. TMP/SMX was added to the drinking water to prevent opportunistic bacterial infections during immunosuppressive drug dosing. Hydration and nutrition of all groups following challenge were supported with DietGel 31 M (Clear H_2_0).

### Anti-NXT-2 antibody functionality: biofilm inhibition and opsonophagocytic killing

To assess functional activity of anti-NXT-2 antibodies, we tested inhibition of *C. albicans* biofilm production. 2 × 10^5^*C. albicans* cells in 95 μL were added to a 96-well plate containing 5 μL of heat-inactivated plasma from CA.KEX1-, NXT-2-, or SHAM-vaccinated mice or NHPs per well. Plates were incubated for 24 hours at 37°C in a humidified chamber, wells were washed twice in PBS, and adhesion was measured by XTT assay (490 nm) (36).

To evaluate antibody-mediated enhancement of phagocytosis and killing, murine macrophages (RAW 264, Sigma–Aldrich, Inc.) were cultured at 37°C in 5% CO_2_ in RPMI-1640 (Irvine Scientific) with 10% FBS, 50 mM β-mercaptoethanol (Thermo Fisher Scientific), and 1% penicillin, streptomycin, and glutamine (Gemini BioProducts). Macrophages were activated by treatment with 1 ng/mL LPS (Sigma–Aldrich, Inc.) for 24 hours and harvested. At the same time, *A. fumigatus* was grown at 37°C for 7 days on minimal media plates (33) and *C. albicans* was grown overnight at 30°C in YPD broth. At a concentration of 2 × 10^5^ cells/mL, 95 μL of *Aspergillus* or *Candida* culture were added to a 96-well microtiter plate with 10 μL of plasma from mice immunized with AF.KEX1, CA.KEX1, NXT-2, or PBS. Plates were incubated at 37°C for 15 minutes, after which activated macrophages were added to each well at a ratio of 1:1 (macrophages: fungal cells). After 2 hours of incubation with gentle shaking, the contents of the wells were quantitatively plated on minimal media (*Aspergillus)* and YPD (*Candida*) agar plates. The % killing of each fungus was calculated as previously described (36), using the following formula: 1- [CFUs from tubes with (mouse plasma + fungi + macrophages)/average CFU in tubes with (fungi + macrophages)] (36).

Hyperimmune plasma (IgG RET 5.64 × 10^5^ to 8.78 × 10^5^) was utilized for all functionality assays. Mouse plasma was collected 28 days following immunization with NXT-2, CA.KEX1, AF.KEX1, or PBS and TiterMax. NHP plasma was collected 2 weeks following a second immunization with NXT-2 or PBS and alum, as described below. Plasma antibodies were confirmed to be predominantly IgG by ELISA (140:1 IgG: IgM; not shown).

### Immunization and SIV infection in NHPs

A total of three groups of rhesus macaques were used to evaluate the efficacy of NXT-2 immunization against *Pneumocystis* infection following SIV infection. Group 1 (*n* = 7) was intramuscularly immunized with 100 μg of recombinant NXT-2 and aluminum hydroxide (Imject Alum, Thermo Scientific) mixed in a 1:1 ratio. Group 2 (*n* = 8) received a sham inoculation with PBS and aluminum hydroxide, and Group 3 (*n* = 8) did not receive any vaccination. Animals were subsequently rested for 8 weeks, after which NXT-2-immunized animals were boosted with 100 μg of NXT-2 + alum and sham-immunized animals were sham inoculated as described above. NXT-2-immunized macaques were bled 1 week post vaccination (1wpv1) and boost (1wpv2) while sham-immunized animals were bled 2wpv1 and 2wpv2 to assess vaccine responses. Following an 8-week rest period from boost, all macaques were infected intravenously with SIV ΔB670, as previously described (41). Viral infection (gag RNA copies/mL plasma) was monitored at weekly time points for 4 weeks and monthly thereafter to 36 weeks post infection. Immunologic parameters described below were monitored at monthly intervals after infection.

### *Pneumocystis macacae* challenge and diagnosis of infection

As *Pneumocystis* (Pc) cannot be reliably cultured in vitro, challenge of NHPs was performed via natural airborne transmission by cohousing these animals with animals coinfected with SIV and PCR-confirmed Pc, as described previously (27, 32, 42). Following SIV infection, Pc infection status was evaluated at monthly intervals by polymerase chain reaction (PCR) analysis of bronchoalveolar lavage fluid (BALF) samples, as described (27, 30, 42–44). To control for the DNA quality in BALF samples, PCR for detection of β-globin was also performed (27, 30, 42, 43, 45). Samples that were negative by PCR were further evaluated for pulmonary *Pneumocystis* pulmonary colonization, defined as detection of Pc DNA in BALF when amplified again in the nested round of PCR, as described previously (27, 30, 33, 44). Chronic colonization was defined as two or more consecutive monthly BALF samples that were positive by nested PCR. During coinfection with SIV and *Pneumocystis*, animals exhibiting evidence of end-stage AIDS (i.e. persistent anorexia, weight loss of > 20%, or symptoms of opportunistic infections) were euthanized. All other animals were euthanized at study termination (i.e. 36 weeks after SIV infection). A total of two NXT-2- and two sham-immunized animals were removed from assessment of Pc infection due to evidence of prolonged SIV control defined by an undetectable viral load and a CD4^+^ T cell count within normal range (> 500/uL).

### Flow cytometry

Peripheral blood and BALF cells from NHPs were isolated, washed, stained, and analyzed as previously described (30). Fixed, stained cells were acquired on an LSRII flow cytometer (BD Biosciences) and analyzed using FlowJo analysis software (BD Biosciences). Plasmablasts were identified as CD138 + CD27hiCD19 + CD20 + IgD–IgM- and other cell populations were identified as previously described (30). Cytometry experiments included fluorescence-minus-one controls and doublet cells were excluded from analyses based on forward scatter-A and forward scatter-H. Lymphocyte population was gated using forward and side scatter. Single-stained Ultra Comp ebeads (ThermoFisher Scientific) were used to compensate for spectral spillover.

### Statistical analysis

All statistical analyses were performed using GraphPad Prism (GraphPad Software). Survival curves were analyzed by Mantel–Cox test between NXT-2-, species-specific KEX1-, and sham-immunized animals. Fungal burden, mean survival time, biofilm inhibition, changes in opsonophagocytic killing, and memory cell populations were analyzed by one-way ANOVA with Tukey correction. Weight and temperature changes in mice, and CD4^+^ T-cell count, SIV viral load, and IgG titer in macaques were analyzed using repeated measures mixed modeling. Post hoc analysis of the significant group by time interaction was performed based on Fisher’s least significant difference procedure for pairwise differences. Differences in sex were assessed by Mantel–Cox for survival and Mann–Whitney for fungal burden across the full roster in challenge studies, as there were no notable associations between sex and vaccine status. Mann–Whitney tests were also utilized for comparison of preselected timepoints of the study baseline and 1 week post vaccine two (1wpv2) for Th1/Th2 analysis.

## Results

### NXT-2 immunization generates cross-reactive antifungal antibodies

**Fig. 2.**
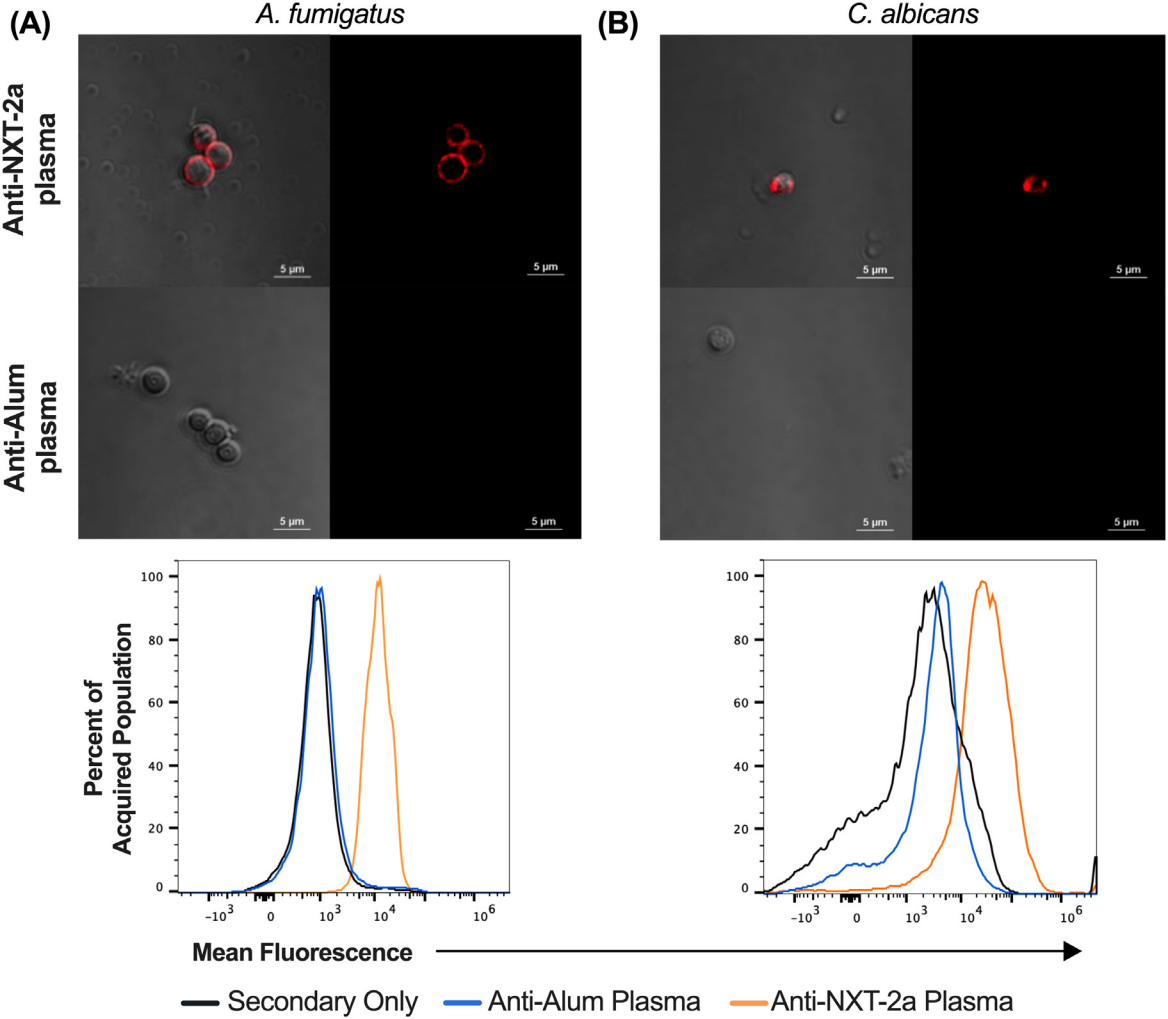
Anti-NXT-2 antibodies bind to the surface of fungal pathogens. (A) *Aspergillus fumigatus* and (B) *Candida albicans* were incubated with plasma from NXT-2a- and SHAM-vaccinated mice and Rhodamine Red antimouse IgG. Upper panels, confocal microscopic images; lower panels, mean fluorescence intensity by flow cytometry.

Recombinant protein vaccines comprising the KEX1 regions of *P. jirovecii* and *A. fumigatus* have been demonstrated to be protective in animal models of their respective fungal infections (30, 33). Based upon these KEX1 sequences, as well as those from *C. albicans* and *C. neoformans*, we generated a pan-fungal consensus sequence (NXT-2; Fig. 1A). The consensus peptide, NXT-2, was expressed in *E. coli*. We immunized NHPs with a version of the NXT-2 protein without the linker or histidine tag (NXT-2a) in order to accurately assess protein cross-reactivity without nonspecific construct recognition (Fig. 1B). Anti-NXT-2a antibodies recognize the species-specific recombinant KEX1 peptides of *A. fumigatus,P. jirovecii,C. albicans,H. capsulatum,C. immitis,M. circinelloides,C. auris*, and *C. neoformans*. The relative intensity of cross-reactivity of the anti-NXT-2a antibodies assessed by western blotting and quantified by ImageJ correlates with the degree of sequence identity between the consensus NXT-2 sequence and the species specific-KEX1 peptides (*P* = 0.0009, Fig. 1C). Further, anti-NXT-2a antibodies bind to the surface of fungal pathogens, including *A. fumigatus* (Fig. 2A) and *C. albicans* (Fig. 2B).

### Immunization with NXT-2 and AF.KEX1 immunization reduces *Aspergillus*-associated morbidity and mortality in immunosuppressed mice

**Fig. 3.**
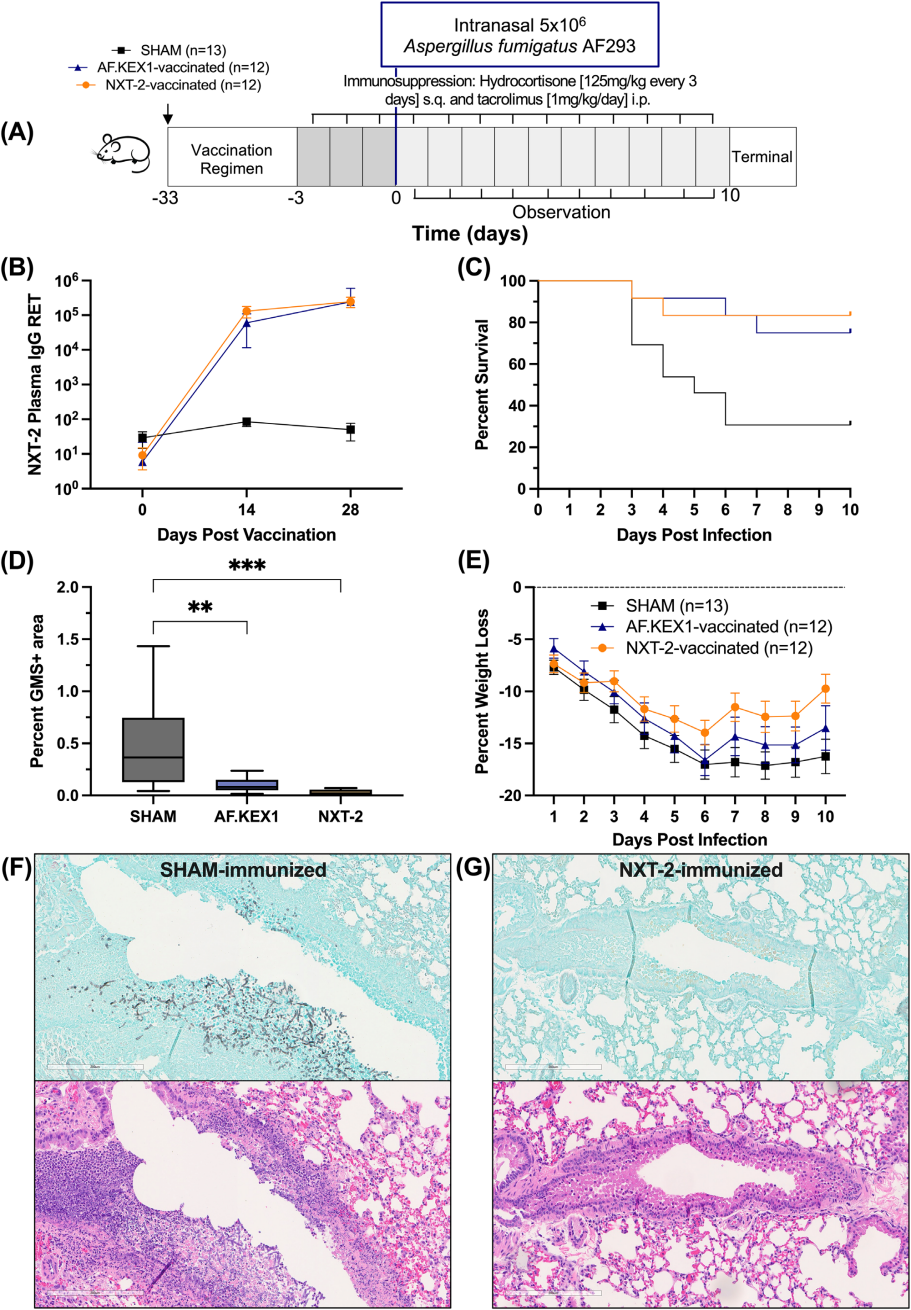
NXT-2 vaccination reduces invasive pulmonary aspergillosis in a model of solid organ transplantation. (A) Aspergillosis challenge study design. Vaccination is indicated with arrows. BALB/c mice were immunized with NXT-2 + TiterMax, AF.KEX1 + TiterMax, or PBS + TiterMax 28 days prior to immunosuppression with hydrocortisone and tacrolimus. (B) NXT-2 plasma IgG reciprocal endpoint titers (RET) in mice immunized with NXT-2 + TiterMax or PBS + TiterMax. Presented RET is against the antigen with which mice were vaccinated. SHAM data is an average of anti-NXT-2 and anti-AF.KEX1 RET. (C) Survival curve of NXT-2- and AF.KEX1-immunized animals, compared with SHAM. NXT-2- and AF.KEX1-immunized animals were significantly protected from developing aspergillosis, compared with mock-immunized controls (*P = 0.0118; *P = 0.0172, by Mantel–Cox test). (D) Quantification of fungal burden in a solid organ transplant model by %GMS + area. (E) Percent weight loss 10 days from *Aspergillus* challenge with significant differences between NXT-2-vaccinated and Sham at 7-, 8-, and 10-days post infection. Data represents the mean ± SD. Differences in weight loss were analyzed by repeated measures mixed modeling. (F) GMS and H&E staining of lung tissue following SHAM or (G) NXT-2 immunization and *Aspergillus* challenge (10x magnification). ** < 0.01, ^***^< 0.001.

A summary of the experimental design is shown in Fig. 3(A). In NXT-2-immunized CD-1 mice, mean plasma anti-NXT-2 IgG reciprocal endpoint titers (± SD) peaked 28 days following the immunization (Fig.   3B; 4.64 × 10^5^ ± 2.92 × 10^5^). These antibody titers are comparable to those achieved with AF.KEX1 immunization (2.44 × 10^5^ ± 3.52 × 10^5^) (33). As a further evaluation of the NXT-2 vaccine candidate in this model, we compared the results of this study to that of species-specific AF.KEX1, which was previously shown to be protective in this model (33). Following immunosuppression and *A. fumigatus* challenge, 2 of 12 (16.7%) mice vaccinated with NXT-2 were euthanized for aspergillosis-related endpoints compared 9 of 13 (69.2%) sham-immunized cohort (Fig. 3C; *P =* 0.0118). Protection level in the NXT-2-immunized group was comparable to the AF.KEX1-immunized (*P* = 0.667). The lung fungal burden in NXT-2-immunized mice was significantly lower than in the sham-immunized cohort (Fig. 3D; *P =* 0.0003) and similar to that from the AF.KEX1-vaccinated cohort (*P =* 0.8143). In addition to similar reductions in mortality compared to AF.KEX1-vaccinated mice, NXT-2-vaccinated mice also demonstrated reduced weight loss compared to SHAM- and species-specific AF.KEX1-vaccinated mice that was significant by 7, 8, and 10 days from *Aspergillus* infection (Fig. 3E; *P = 0.0186, *P = 0.0452, and *P = 0.0024). There were no significant differences in mortality or fungal burden between sexes (not shown; *P =* 0.0766 and *P =* 0.1564). By GMS and H&E staining, the lung pathology of the SHAM-immunized mice demonstrated increased cellular infiltrate and granuloma formation, with the latter associated with *Aspergillus* hyphal formation, compared to the lung pathology of NXT-2-immunized mice (Fig. 3F and G).

### Immunization with NXT-2 and CA.KEX1 reduces morbidity and mortality from invasive candidiasis in immunosuppressed mice

**Fig. 4.**
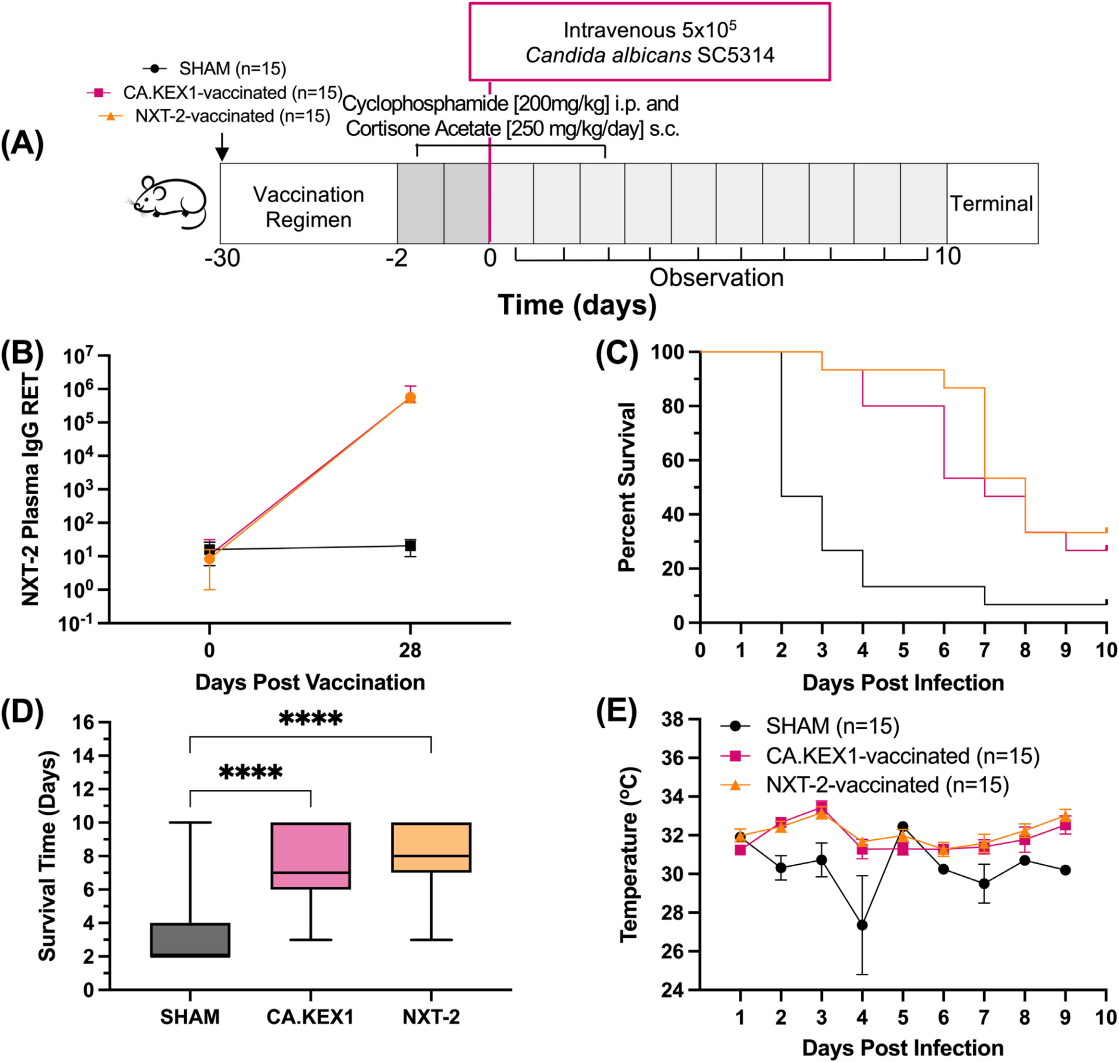
NXT-2 vaccination reduces invasive candidiasis in a model of neutropenia. (A) Invasive candidiasis challenge study design. Vaccination is indicated by an arrow. CD-1 mice were immunized with NXT-2 + TiterMax, CA.KEX1 + TiterMax, or PBS + TiterMax 28 days prior to immunosuppression with cortisone acetate and cyclophosphamide. (B) Plasma IgG reciprocal endpoint titers (RET) in mice immunized with NXT-2 + TiterMax, CA.KEX1 + TiterMax, or PBS + TiterMax. Presented RET is against the antigen with which mice were vaccinated. SHAM data is an average of anti-NXT-2 and anti-CA.KEX1 RET. (C) Survival curve of NXT-2 and CA.KEX1-immunized animals, compared with SHAM. Both NXT-2- and CA.KEX1-immunized animals had a significant reduction in *Candida*-associated mortality, compared with sham-immunized controls (*P* = 0.0001; *P* = 0.0015, by Mantel–Cox test). (C) Average survival time by group in days, analyzed by one-way ANOVA. (D) Body temperature to 10 days from *Candida* challenge with significant differences between NXT-2-vaccinated and SHAM at 2-, 3-, and 4-days post infection. Data represents the mean ± SD. Differences in weight loss were analyzed by repeated measures mixed modeling. ^****^ < 0.0001.

A summary of the experimental design is shown in Fig. 4(A). In these experiments, we tested the protective efficacy of the species-specific *C. albicans* KEX1 subunit vaccine (CA.KEX1) and NXT-2. Mean plasma anti-NXT-2 IgG reciprocal endpoint titers (± SD) peaked 28 days following the immunization with NXT-2 + TiterMax (Fig. 4B; 5.68 × 10^5^ ± 5.66 × 10^5^). These antibody titers are comparable to those achieved with CA.KEX1 immunization (Fig. 4B; 5.64 × 10^5^ ± 6.65 × 10^5^). In total, 14 of 15 (93.3%) sham-immunized cohort were euthanized for *Candida*-related endpoints while 10 of 15 (66.7%) mice vaccinated with NXT-2 (*P* = 0.0001) and 11 of 15 (73.3%) mice vaccinated with CA.KEX1 (Fig. 4C; *P* = 0.0015) met the same endpoints. In addition to increased overall survival, the mean survival time of the vaccinated animals was significantly greater in mice immunized with CA.KEX1 (7.1 days, *P* < 0.0001) and NXT-2 (7.9 days, *P* < 0.0001), compared to a mean survival of 3.3 days in the control group (Fig. 4D). CA.KEX1- and NXT-2-immunized mice exhibited more stable body temperatures over the course of the study, significantly higher than controls at 2 (*P* < 0.0001), 3 (*P* = 0.0016), and 4 (*P* < 0.0001) days post infection (Fig. 4E). There were no significant differences in mortality or survival time between sexes (not shown; *P* = 0.1679, *P* = 0.0910).

### Vaccine-induced antibodies reduce biofilm formation of *C. albicans*

**Fig. 5.**
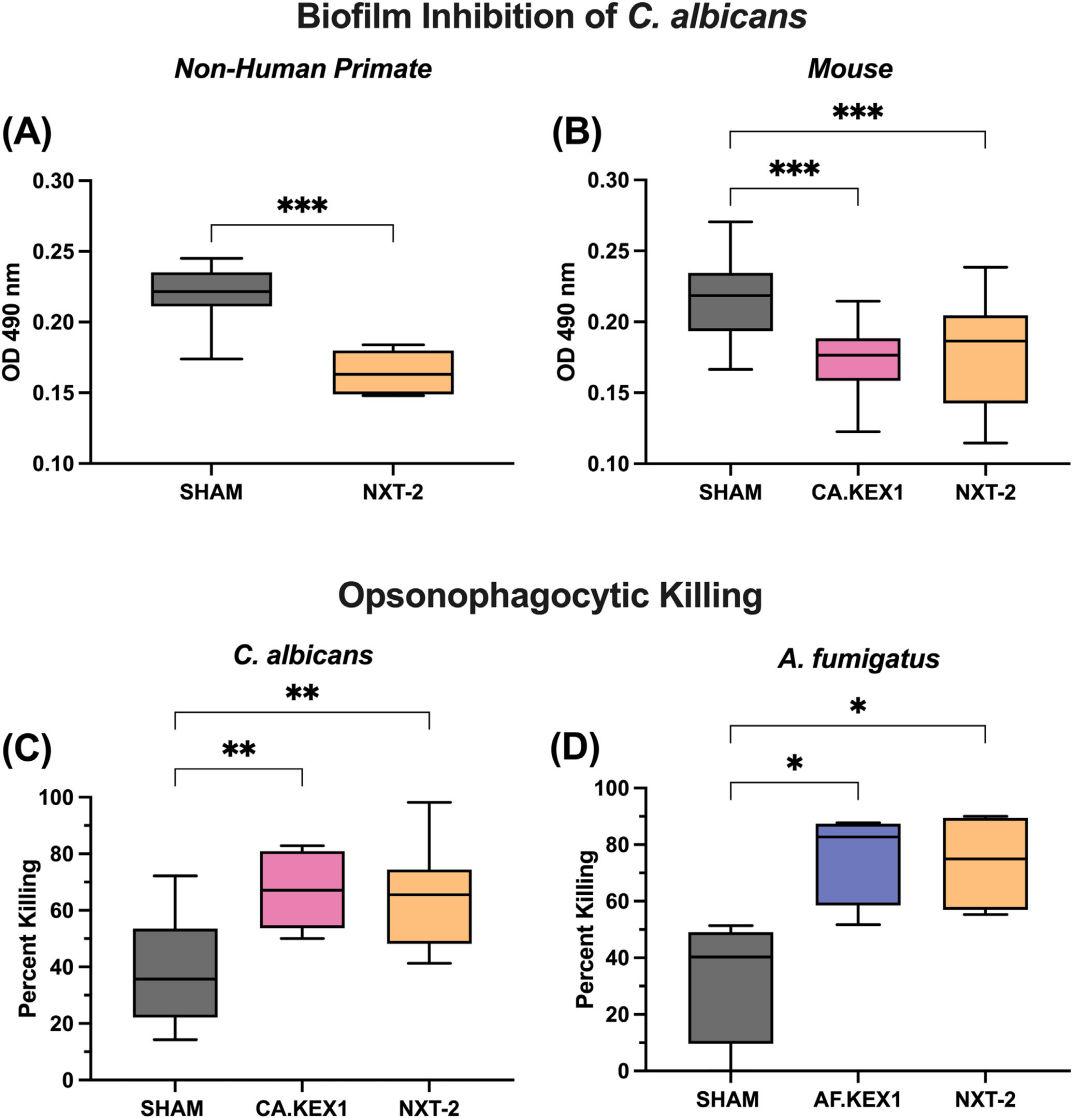
Anti-NXT-2 antibodies inhibit biofilm formation and increase opsonophagocytic killing. *Candida albicans* biofilms were incubated with heat-inactivated plasma from (A) CA.KEX1-, NXT-2-, or SHAM-immunized mice and (B) NXT-2- or SHAM-immunized rhesus macaques, with reduced formation in wells with plasma from vaccinated mice and NHPs. (C) and (D) Opsonophagocytic killing of *C. albicans* and *A. fumigatus* by macrophages was evaluated in presence of plasma from CA.KEX1-, AF.KEX1-, NXT-2-, and SHAM-immunized mice. Plasma from CA.KEX1-, AF.KEX1-, and NXT-2-immunized mice significantly enhanced fungal killing compared to SHAM. Results are presented relative to macrophage killing of fungi without any added plasma. Statistical significance was determined by Mann–Whitney tests and one-way ANOVA. ^*^≤ 0.05, ** < 0.01, and *** < 0.001.

Treatment of *C. albicans* with hyperimmune plasma from NXT-2-vaccinated NHPs significantly reduced biofilm formation (Fig. 5A, *P* = 0.0006) compared to plasma from sham-vaccinated animals. Plasma from mice vaccinated with either CA.KEX1 + TiterMax or NXT-2 + TiterMax also significantly reduced biofilm formation (Fig. 5B, CA.KEX1, *P* = 0.0004; NXT-2, *P* = 0.0007) compared to plasma from sham-vaccinated mice.

### Vaccine-induced antibodies enhance opsonophagocytic killing of *C. albicans* and *A. fumigatus* by macrophages

Incubation of *C. albicans* with hyperimmune plasma from CA.KEX1- or NXT-2-immunized mice increased opsonophagocytic killing of *C. albicans* (Fig. 5C; CA.KEX1, *P* = 0.0018; NXT-2, *P* = 0.0037) by murine macrophages. Further, incubation of *A. fumigatus* with hyperimmune plasma from AF.KEX1- or NXT-2-immunized mice increased fungal killing (Fig. 5D; *P* = 0.0002, *P* < 0.0001) compared to sham-vaccinated.

### Immunization with NXT-2 reduces *Pneumocystis* infection and colonization in SIV-infected, immunosuppressed NHPs

**Fig. 6.**
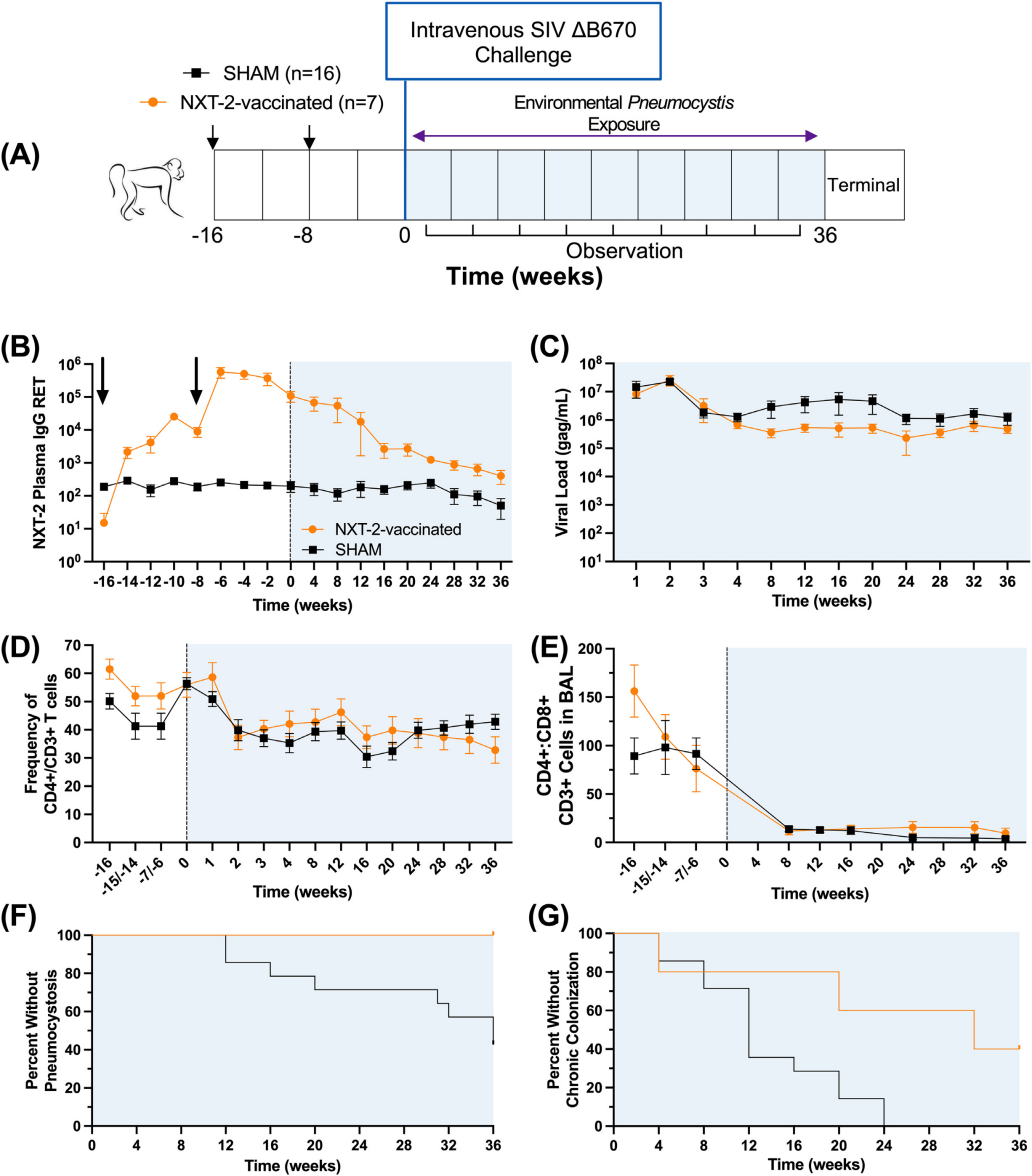
NXT-2 vaccination reduces pneumocystosis in a NHP model of HIV. (A) Pneumocystosis challenge study design. Rhesus macaques were immunized and boosted with NXT-2 + alum or PBS + alum 8 and 16 weeks prior to SIV infection. Vaccination is indicated with arrows. (B) Mean plasma NXT-2-specific immunoglobulin G (IgG) titer, as determined by enzyme-linked immunosorbent assay in rhesus macaques immunized with NXT-2 + alum or PBS + alum, following vaccination and through 36 weeks of SIV infection. (C) Viral load, (D) peripheral blood CD4^+^ T cell frequency, and (E) the ratio of CD4^+^ T cells to CD8^+^ T cells in the BALF of SIV-infected rhesus macaques. (F) Pneumocystosis (defined by Wakefield positive PCR in BALF in NXT-2- and SHAM-immunized rhesus macaques. NXT-2-immunized NHPs were significantly protected from developing Pc infection, compared with mock-immunized controls (*P = 0.0451, by Mantel–Cox test). (G) Chronic colonization (defined as two or more consecutive monthly nested positive PCR results in the BALF) in all cohorts. NXT-2-vaccinated NHPs had reduced colonization compared to SHAM-immunized (*P = 0.0191) NHPs. BALF, bronchoalveolar lavage fluid. Data represents the mean ± SD. Differences in T cells and viral load were analyzed by repeated measures mixed modeling.

A summary of the experimental design is shown in Fig. 6(A). In NXT-2-immunized macaques, the mean plasma anti-NXT-2 IgG reciprocal endpoint titers (± SD) peaked 2 weeks following the boost with NXT-2 + alum (Fig. 6B; 5.78 × 10^5^ ± 5.37 × 10^5^). At the time of SIV infection, plasma anti-NXT-2 IgG titers had declined somewhat but were significantly higher in NXT-2-immunized animals (1.08 × 10^5^ ± 1.04 × 10^5^), compared with sham-immunized macaques (197.13 ± 199.91; **P = 0.0048). Plasma anti-NXT-2 titers of vaccinated NHPs remained significantly higher than the sham-immunized cohort over the course of SIV infection, up to 32 weeks post infection. The anti-NXT-2 peak antibody titers and titers following SIV-induced immunosuppression were comparable to those previously achieved with species-specific PC.KEX1 immunization (30).

We have previously characterized the natural course of *Pneumocystis* infection in macaques and demonstrated that they are susceptible to Pc colonization and infections as a consequence of SIV-induced immunosuppression (27, 32, 42). SIV infection and immunosuppression were evaluated by plasma viral load and CD4^+^ T cell levels. There was no significant difference in viral loads between the vaccinated and unvaccinated groups (*P =* 0.9929; Fig. 6C). The frequency of circulating CD4^+^ T cells declined within the first 2 weeks of infection and remained depressed throughout the duration of SIV infection, but the trend was similar between groups (*P* = 0.5786; Fig. 6D). The ratio of CD4^+^ to CD8^+^ cells in the BALF decreased after SIV infection in both groups, again in all cohorts (*P* = 0.2957), which is consistent with persistent SIV infection. (Fig. 6E) (46).

After immunization and SIV infection, all groups were exposed to natural transmission of *Pneumocystis macacae* by cohousing in rooms with evidence of circulating Pc, as previously described (27, 32, 42). None of the animals had evidence of *Pneumocystis* colonization at the time of SIV infection, but Pc colonization was detectable in some animals by 4 weeks following SIV infection when peripheral blood CD4^+^ T cells had declined (27, 32, 42). At study termination (36 weeks after challenge), 8 of 14 (57.1%) of the sham macaques had pneumocystosis, while no NXT-2-immunized animals tested positive (*P* = 0.0451). This indicates that animals immunized with NXT-2 were protected from developing pneumocystosis (Fig. 6F). These results were consistent with previous findings of protection against Pc infection with PC.KEX1 vaccination (30).

In an assessment of chronic Pc colonization, we previously found that while KEX1 vaccination in both SIV- (30) and drug-immunosuppressed (47) models prevented the development of pneumocystosis, it did not prevent colonization. In the present study, all animals in the control group (*n* = 14) became chronically colonized following SIV infection, as defined by two consecutive months of nested positive BALF PCR results (Fig. 6G). In contrast, only three of the five (60%) NXT-2-vaccinated animals were chronically colonized after natural transmission over the course of 36 weeks of SIV, which was a significant reduction compared to sham-immunized (*P = 0.0191) macaques.

### Humoral and cellular immune responses to NXT-2 vaccination

**Fig. 7.**
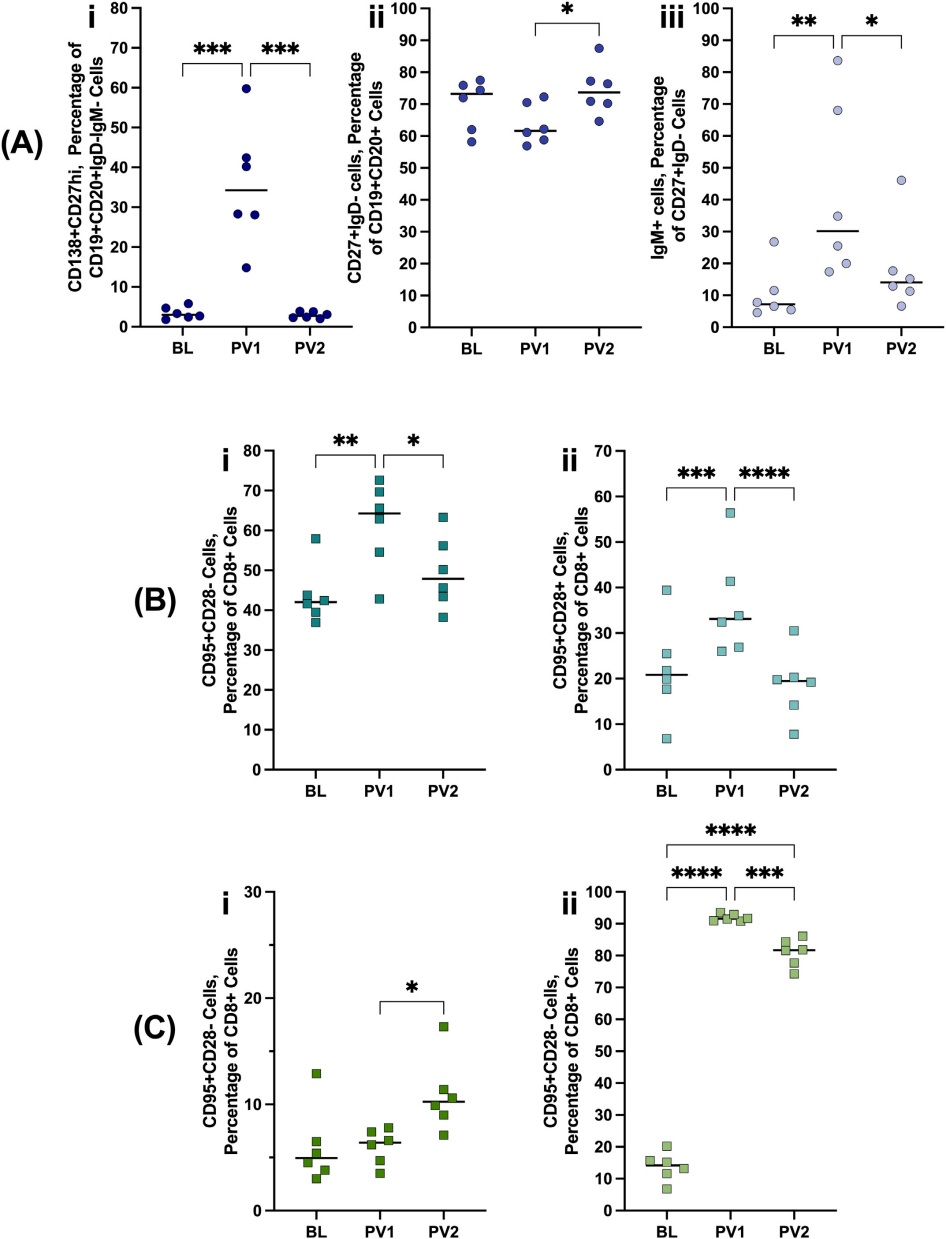
NXT-2 vaccination-associated memory responses. (A) Development of B cell memory was analyzed at baseline, and 1 week following vaccination (PV1) and boost (PV2) with NXT-2 + alum in rhesus macaques. The cell populations analyzed here are the frequency of (i) plasmablasts (CD138 + CD27hiCD19 + CD20 + IgD–IgM-), (ii) switched memory (CD27 + IgD-CD19 + CD20+), and (iii) percentage of switched memory (CD27 + IgD-CD19 + CD20+) B cells that are IgM+ . Memory T-cell populations were also analyzed in the peripheral blood (B) and BALF (C), including the frequency of (i) effector memory and (ii) central memory T cells. Changes in cell populations over time were analyzed by one-way ANOVA. * ≤ 0.05, ^**^< 0.01, and *** < 0.001.

The development of immunologic memory was assessed through identification of peripheral blood plasmablasts (CD138 + CD27hiCD19 + CD20 + IgD–IgM-) and switched memory B cells (CD27 + IgD-CD19 + CD20+). The immature plasmablast population significantly increased in frequency at 1 week post vaccination 1 (1wpv1) before resolving to baseline levels (Fig. 7Ai; ***P = 0.0003). Accordingly, switched memory B cells significantly increased with the second immunization (Fig. 7Aii; *P = 0.0264). Within this memory B-cell population, IgM+ cells significantly increased with the first immunization before resolving to baseline levels (Fig. 7Aiii; *P =* 0.0046). Peripheral blood T cell effector (Fig. 7Bi; CD95 + CD28-CD8 + CD3+) and central memory (Fig. 7Bii; CD95 + CD28 + CD8 + CD3+) cells significantly increased with the first immunization before returning to baseline levels (**P = 0.0024; ***P = 0.0001). Memory T cell responses in the lung environment were mixed with central memory also increasing substantially with the first immunization but additionally remaining elevated following the boost (Fig. 7Cii; ***P < 0.0001). The change in BALF effector memory T cell population was captured 1 week after the boost, which is comparable to the reported kinetics of T cell memory establishment in the lung (48).

### NXT-2 and circulating CD4^+^ T-Helper type 1 (Th1) and Type 2 (Th2) cell populations

**Fig. 8.**
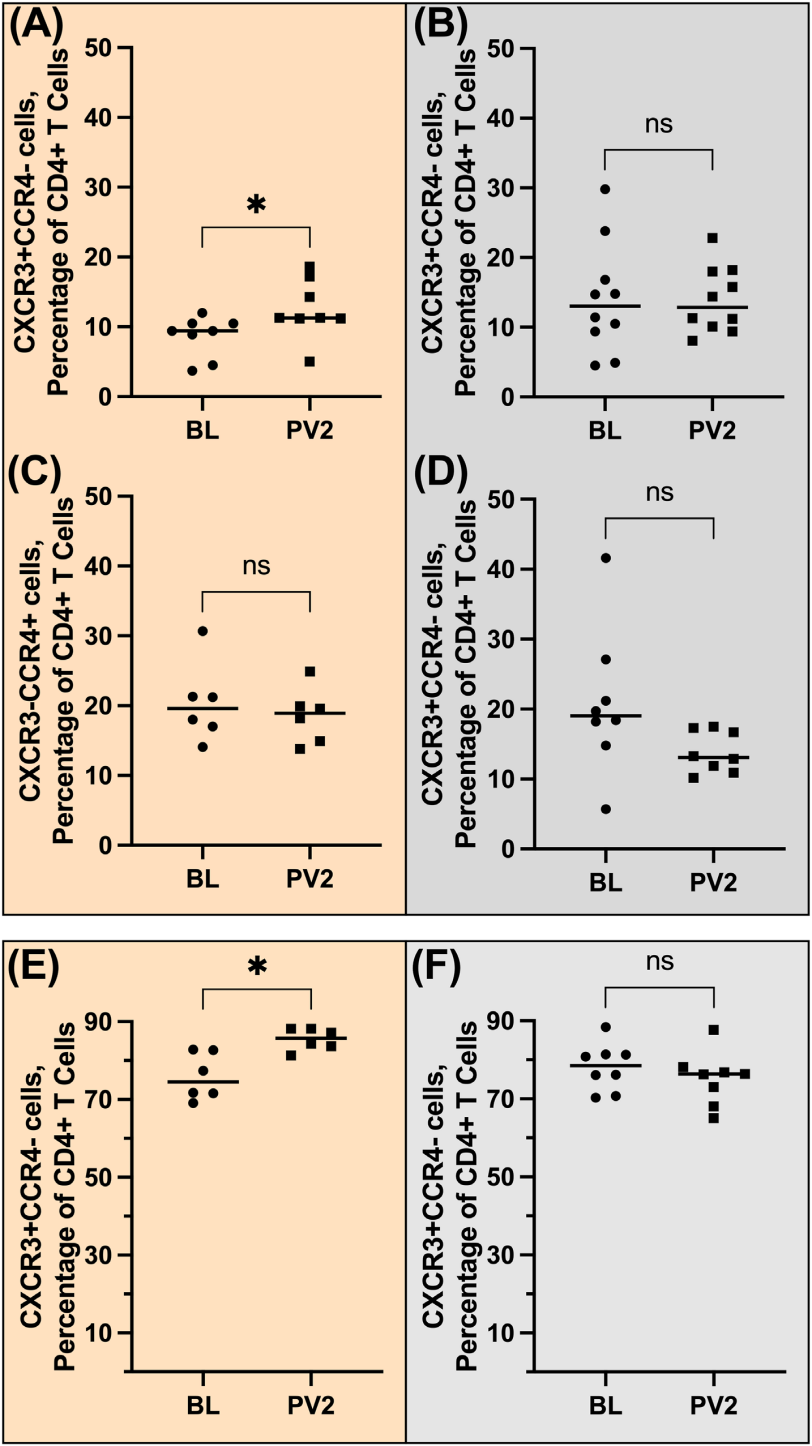
T-helper type frequencies in NXT-2 vaccination. (A)–(D) Peripheral blood and (E) and (F) BALF lymphocytes from rhesus macaques were analyzed by flow cytometry for surface markers associated with either Th1 (CXCR3^+^CCR4^−^) or Th2 (CCR4^+^CXCR3^−^) CD4^+^cells and expressed as a percentage of total peripheral blood CD4^+^ T cells. Frequencies of T-helper populations for NXT-2-immunized animals are given in panels (A), (C), and (E); frequencies of T-helper populations for sham-immunized animals are given in panels (B), (D), and (F). Wilcoxon tests were used to compare samples collected at baseline (BL) and following vaccination two (PV2) timepoints. * ≤ 0.05.

NXT-2-immunized NHPs were evaluated for T-helper cell response skewing following immunization. A significant increase in frequency of peripheral blood Th1 cells was found at in NXT-2-immunized macaques (Fig. 8A; **P = 0.0078); however no significant change in the frequency of Th1 cells was seen in sham-immunized animals post boost (Fig. 8B; *P =* 0.4922). At the same timepoint, the frequency of CD4^+^ Th2 cells was similar to the frequency at baseline in NXT-2-immunized animals (Fig. 8C; *P =* 0.0781) and sham-immunized animals (Fig. 8D; *P =* 0.4375). In the BALF, there was a significant increase in frequency of Th1 cells in NXT-2-immunized macaques (Fig. 8E; *P = 0.0312) while no change was observed in sham-immunized animals (Fig. 8F; *P =* 0.4609). In a direct comparison of Th1 and Th2 phenotypes, NHPs immunized with NXT-2 + alum did not have significant difference in the frequency of Th1 and Th2 cells in the peripheral blood and mice immunized with NXT-2 + TiterMax did not have a significant difference in Th1 and Th2 antibody phenotypes (Figure S2).

## Discussion

A safe, effective, and broadly protective pan-fungal vaccine to prevent or ameliorate IFIs would be highly beneficial in reducing morbidity and mortality in individuals with high-risk conditions. This would include individuals scheduled to begin immunosuppressive therapies as they are often susceptible to infections by multiple fungal pathogens, principally *Aspergillus,Candida,and Pneumocystis* species. Other antigens have been proposed in “pan-fungal” vaccine strategies, including the cell wall protein β-1,6-glucan (49) and Calnexin (50). However, no pan-fungal or single-pathogen antifungal vaccines have been approved for clinical use.

This study is the first to utilize a recombinant protein-based pan-fungal vaccine candidate in multiple models of invasive fungal disease, including aspergillosis, candidiasis, and pneumocystosis. Our work with NXT-2 builds upon previous research utilizing recombinant KEX1 proteins as vaccine candidates to achieve protection against fungal challenge (30, 33). We found that anti-NXT-2 antibodies are cross-reactive and recognize recombinant KEX1 proteins derived from *A. fumigatus,C. albicans*, and *P. jirovecii*, two of which have been previously demonstrated to be protective in their respective models of invasive fungal disease (30, 33). Vaccination with NXT-2 significantly reduces morbidity and mortality in severely immunosuppressed animal models of aspergillosis, candidiasis, and pneumocystosis compared to controls. We further found that NXT-2 vaccination improved some morbidity outcomes beyond both sham- and species-specific KEX1 vaccinations.

NXT-2 is highly immunogenic in both mice and NHPs. Anti-NXT-2 antibodies appear to function by surface binding to the fungi and promoting opsonophagocytic killing. Additionally, anti-NXT-2 antibodies inhibited biofilm formation, a major virulence factor of *C. albicans*. Anti-NXT-2 plasma from both NHPs and mice increased inhibition of *C. albicans* biofilm formation, indicating comparable vaccine performance across species and vaccine-adjuvant formulations.

In the murine model of invasive aspergillosis, we found NXT-2 to be associated with a significant reduction in mortality, lung fungal burden, and weight loss. The protection associated with NXT-2 immunization was comparable to that previously established with the species-specific vaccine candidate AF.KEX1 (33). In a murine model of invasive candidiasis, a single immunization with NXT-2 significantly reduced mortality and more than doubled the average survival time of immunosuppressed mice following lethal challenge with *C. albicans*. Although this is a highly aggressive immunosuppressive regimen and a high dose, intravenous fungal challenge, the reduction in mortality and increased survival time supports further exploration and optimization of NXT-2 in this model of invasive candidiasis. Further, the increased length of survival time associated with NXT-2 vaccination could allow crucial hours or days that are often needed to diagnose and begin antifungal treatment (51).

The efficacy of the NXT-2 vaccine was further tested in a NHP model of HIV and *Pneumocystis* coinfection. As we previously reported, immune suppression associated with SIV infection leads to a natural susceptibility of NHPs to Pc infection by environmental exposure (27, 32, 42). In the present study, we demonstrated that immunization with NXT-2 resulted in robust and durable plasma IgG titers and significantly reduced the incidence of Pc infection compared to sham-immunized animals. NXT-2 vaccination was also associated with a reduction in the frequency of chronic Pc colonization compared to SHAM-immunized animals. Persistent Pc colonization has previously been identified as a cofactor of COPD (52, 53) and may contribute to severity of symptoms in asthma patients (54). Reduction in chronic colonization achieved with NXT-2 supports further exploration of this novel strategy for the improvement of long-term outcomes in these patient populations.

In addition to demonstration of protective efficacy against the *A. fumigatus,C. albicans,and P. macacae*, we demonstrated that anti-NXT-2 polyclonal antibodies are cross-reactive with recombinant KEX1 homologs from other fungal pathogens, including *C. auris,C. immitis,C. neoformans,H. capsulatum*, and *M. circinelloides*. This cross-reactivity indicates additional pathogens to which NXT-2 immunization may potentially provide some protection.

Interrogation of the immune response to NXT-2 immunization in macaques revealed a significant increase in the circulating memory B- (55) and T-cell (56) populations. Plasmablasts, as well as effector and central memory T-cell populations peaked following the first vaccination and switched B-cell memory peaked following the boost. In addition, we did not observe significant Th2 skewing with an alum-based vaccination and observed significant increases in the frequency of Th1 CD4^+^ cells with the vaccine boost in both peripheral blood and BALF that were not echoed in Th2 CD4^+^ cell compartment. These findings further support the hypothesis that vaccination with NXT-2 will induce a robust and durable humoral response.

It is worth noting that the broad efficacy of a kexin-based vaccine strategy is somewhat surprising. Kexin is primarily associated with the trans-Golgi network; however, anti-KEX1 and anti-NXT-2 antibodies detected this protein on the surface of multiple fungal pathogens, including *A. fumigatus* and *C. albicans*. It is possible that the epitopes recognized by protective antibodies elicited by the species-specific KEX1 and NXT-2 immunizations are recognizing epitopes present in a surface protein that have shared sequence homology with the internal kexin peptidase. Additional explanations include some transmembrane presentation of kexin or surface localization due to trafficking of intracellular molecules through the cell wall, as has been described in *Aspergillus* (57). Alternatively, recognition could occur as dead or dying fungi shed proteins with subsequent adherence to the surface of viable cells. Immune recognition of intracellular fungal proteins is also not unprecedented. For example, *Histoplasma* heat shock proteins (58) and a calnexin protein fragment (50) have been located on the cell surface and immunization with these proteins was associated with protective immunity.

While there several potential explanations, the efficacy of anti-NXT-2 antibodies in the surface binding to the fungi, promoting opsonophagocytic killing and inhibiting biofilm formation, and induced protection in multiple in vivo models of fungal disease indicate some surface presentation and consistent immune recognition.

These studies have some limitations as the group sizes for some studies are small; however, all studies were repeated at least once. Additionally, these findings are supported by previous work demonstrating vaccine efficacy with the related antigens, PC.KEX1 (30, 47) and AF.KEX1 (33). In the SIV and Pc coinfection model, animals used were predominantly female, limiting sex analysis. However, there were no significant differences between sexes seen in the other models of invasive fungal disease. *Pneumocystis* challenge was via natural transmission, which more accurately models the likely route by which susceptible patients acquire Pc but lacks the precision of a dose-associated challenge and timing. However, we can demonstrate that protection from Pc in NXT-2-immunized animals was not due to lack of Pc exposure as Pc colonization was present in sham-immunized animals that were housed in the same room.

In summary, we report the development, immunogenicity, and protective efficacy of a pan-fungal vaccine candidate, NXT-2. Immunization of mice and NHPs with NXT-2 induces robust and durable immunity following varied immunosuppressive regimens and fungal challenges against the three most clinically relevant fungal pathogens *Aspergillus, Candida*, and *Pneumocystis*. These results present NXT-2 as novel pan-fungal vaccine candidate that may reduce morbidity and mortality associated with the three most prevalent causes of IFIs and may provide an alternative to long term antifungal prophylaxis in individuals at risk of IFIs. Further research will address the optimal vaccine formulations, dosing, routes, strategies, and additional clinical indications for an invasive fungal vaccine for humans.

## Supplementary Material

pgac248_Supplemental_Files

## Data Availability

All data is included in the manuscript and supporting materials

## Notes

**Competing Interesrt:** K.A.N., E.R., and W.R. are coinventors of inventions related to research reported here. An approved plan is in place with the University of Georgia for managing potential conflicts. The remaining authors declare that the research was conducted in the absence of any commercial or financial relationships that could be construed as a potential conflict of interest.

## References

[1] Bongomin F , Gago S, Oladele RO, Denning DW. 2017. Global and multi-national prevalence of fungal diseases-estimate precision. J Fungi. 3(4):57. 10.3390/jof3040057 PMC5753159 29371573

[2] Rayens E , Norris K. 2022. Prevalence and healthcare burden of fungal infections in the United States, 2018. Open Forum Infect Dis. 9(1): ofab593. 35036461 10.1093/ofid/ofab593 PMC8754384

[3] Kontoyiannis DP et al. 2010. Prospective surveillance for invasive fungal infections in hematopoietic stem cell transplant recipients, 2001-2006: overview of the Transplant-Associated Infection Surveillance Network (TRANSNET) database. Clin Infect Dis. 50(8):1091–1100. 20218877 10.1086/651263

[4] Pappas PG et al. 2010. Invasive fungal infections among organ transplant recipients: results of the Transplant-Associated Infection Surveillance Network (TRANSNET). Clin Infect Dis. 50(8):1101–1111. 20218876 10.1086/651262

[5] Sipsas NV , Kontoyiannis DP. 2012. Invasive fungal infections in patients with cancer in the intensive care unit. Int J Antimicrob Agents. 39(6):464–471. 22337064 10.1016/j.ijantimicag.2011.11.017 PMC3855365

[6] Bishu S et al. 2014. Rheumatoid arthritis patients exhibit impaired *Candida albicans*-specific Th17 responses. Arthritis Res Ther. 16(1):R50. 24513269 10.1186/ar4480 PMC3978747

[7] Martinez-Martinez MU et al. 2012. Invasive fungal infections in patients with systemic lupus erythematosus. J Rheumatol. 39(9):1814–1818. 22707608 10.3899/jrheum.111498

[8] Silva MF et al. 2015. A multicenter study of invasive fungal infections in patients with childhood-onset systemic lupus erythematosus. J Rheumatol. 42(12):2296–2303. 26568586 10.3899/jrheum.150142

[9] Masur H et al. 1981. An outbreak of community-acquired *Pneumocystis carinii* pneumonia: initial manifestation of cellular immune dysfunction. N Engl J Med. 305(24):1431–1438. 6975437 10.1056/NEJM198112103052402

[10] Webb B et al. 2018. Epidemiology and clinical features of invasive fungal infection in a US health care network. Open Forum Infect Dis. 5(8):ofy187. 30151412 10.1093/ofid/ofy187 PMC6104777

[11] Marr KA , Platt A, Tornheim JA et al. 2021. Aspergillosis complicating severe coronavirus disease. Emerg Infect Dis. 27(1):18–25. 33084566 10.3201/eid2701.202896 PMC7774554

[12] Meijer EFJ , Dofferhoff ASM, Hoiting O, Meis JF. 2021. COVID-19-associated pulmonary aspergillosis: a prospective single-center dual case series. Mycoses. 64(4):457–464. 33569857 10.1111/myc.13254 PMC7986084

[13] Pal R , Singh B, Bhadada SK et al. 2021. COVID-19-associated mucormycosis: an updated systematic review of literature. Mycoses. 64: 1452–1459. 34133798 10.1111/myc.13338 PMC8447126

[14] Bongomin F. 2020. Post-tuberculosis chronic pulmonary aspergillosis: an emerging public health concern. PLoS Pathog. 16(8):e1008742. 32817649 10.1371/journal.ppat.1008742 PMC7440622

[15] Vanderbeke L et al. 2018. Invasive pulmonary aspergillosis complicating severe influenza: epidemiology, diagnosis and treatment. Curr Opin Infect Dis. 31(6):471–480. 30299367 10.1097/QCO.0000000000000504

[16] Coste A et al. 2021. The extent of Aspergillosis in critically ill patients with severe influenza pneumonia: a multicenter cohort study. Crit Care Med. 49: 934–942., 33591000 10.1097/CCM.0000000000004861

[17] Rayens E , Norris KA, Cordero JF. 2021. Mortality trends in risk conditions and invasive mycotic disease in the United States, 1999-2018. Clin Infect Dis. 74: 309–318. 10.1093/cid/ciab336 PMC8800183 33876235

[18] GAFFI . Fungal disease frequency. [accessed 2022 Jun 16]. https://gaffi.org/why/fungal-disease-frequency/.

[19] Verweij PE , Chowdhary A, Melchers WJ, Meis JF.. 2016. Azole resistance in *Aspergillus fumigatus*: can we retain the clinical use of mold-active antifungal azoles?. Clin Infect Dis. 62(3):362–368. 26486705 10.1093/cid/civ885 PMC4706635

[20] van der Linden JW et al. 2015. Prospective multicenter international surveillance of azole resistance in *Aspergillus fumigatus*. Emerg Infect Dis. 21(6):1041–1044. 25988348 10.3201/eid2106.140717 PMC4451897

[21] Astvad KMT et al. 2018. Update from a 12-year nationwide fungemia surveillance: increasing intrinsic and acquired resistance causes concern. J Clin Microbiol. 56(4): e01564–17. 29212705 10.1128/JCM.01564-17 PMC5869841

[22] Garvey M , Meade E, Rowan NJ. 2022. Effectiveness of front line and emerging fungal disease prevention and control interventions and opportunities to address appropriate eco-sustainable solutions. Sci Total Environ. 851(Pt 2):158284. 36029815 10.1016/j.scitotenv.2022.158284

[23] Toda M , Beer KD, Kuivila KM, Chiller TM, Jackson BR. 2021. Trends in agricultural triazole fungicide use in the United States, 1992-2016 and possible implications for antifungal-resistant fungi in human disease. Environ Health Perspect. 129(5):55001. 33949891 10.1289/EHP7484 PMC8098123

[24] Pagano L , Mayor S. 2018. Invasive fungal infections in high-risk patients: report from TIMM-8 2017. Future Sci OA. 4(6):FSO307. 30057784 10.4155/fsoa-2018-0019 PMC6060393

[25] Pagano L , Lumb J. 2011. Update on invasive fungal disease. Fut Microbiol. 6(9):985–989. 10.2217/fmb.11.85 21958139

[26] Person AK , Kontoyiannis DP, Alexander BD. 2011. Fungal infections in transplant and oncology patients. Hematol Oncol Clin North Am. 25(1):193–213. 21236398 10.1016/j.hoc.2010.11.013

[27] Kling HM , Shipley TW, Patil S, Morris A, Norris KA. 2009. Pneumocystis colonization in immunocompetent and simian immunodeficiency virus-infected cynomolgus macaques. J Infect Dis. 199(1):89–96. 19014344 10.1086/595297 PMC2622722

[28] Zheng M et al. 2005. CD4^+^ T cell-independent DNA vaccination against opportunistic infections. J Clin Invest. 115(12):3536–3544. 16308571 10.1172/JCI26306 PMC1288835

[29] Wells J , Haidaris CG, Wright TW, Gigliotti F. 2006. Active immunization against *Pneumocystis carinii* with a recombinant *P. carinii* antigen. Infect Immun. 74(4):2446–2448. 16552076 10.1128/IAI.74.4.2446-2448.2006 PMC1418926

[30] Kling HM , Norris KA. 2016. Vaccine-induced immunogenicity and protection against pneumocystis pneumonia in a nonhuman primate model of HIV and pneumocystis coinfection. J Infect Dis. 213(10):1586–1595. 26823337 10.1093/infdis/jiw032 PMC4837913

[31] Gingo MR et al. 2011. Serologic responses to pneumocystis proteins in HIV patients with and without *Pneumocystis jirovecii* pneumonia. J Acquir Immune Defic Syndr. 57(3):190–196. 21372726 10.1097/QAI.0b013e3182167516 PMC3150634

[32] Kling HM , Shipley TW, Patil SP et al. 2010. Relationship of *Pneumocystis jiroveci* humoral immunity to prevention of colonization and chronic obstructive pulmonary disease in a primate model of HIV infection. Infect Immun. 78(10):4320–4330. 20660609 10.1128/IAI.00507-10 PMC2950365

[33] Rayens E et al. 2021. Vaccine-induced protection in two murine models of invasive pulmonary aspergillosis. Front Immunol. 12:670578. 34084170 10.3389/fimmu.2021.670578 PMC8167062

[34] Schneider CA , Rasband WS, Eliceiri KW. 2012. NIH image to ImageJ: 25 years of image analysis. Nat Methods. 9(7):671–675. 22930834 10.1038/nmeth.2089 PMC5554542

[35] Hohl TM et al. 2005. *Aspergillus fumigatus* triggers inflammatory responses by stage-specific beta-glucan display. PLoS Pathog. 1(3):e30. 16304610 10.1371/journal.ppat.0010030 PMC1287910

[36] Singh S et al. 2019. The NDV-3A vaccine protects mice from multidrug resistant *Candida auris* infection. PLoS Pathog. 15(8):e1007460. 31381597 10.1371/journal.ppat.1007460 PMC6695204

[37] Herbst S et al. 2013. A new and clinically relevant murine model of solid-organ transplant aspergillosis. Dis Model Mech. 6(3):643–651. 23264562 10.1242/dmm.010330 PMC3634648

[38] Stolz DJ , Sands EM, Amarsaikhan N, Tsoggerel A, Templeton SP. 2018. Histological quantification to determine lung fungal burden in experimental aspergillosis. J Vis Exp. 9(133): 57155. 10.3791/57155 PMC5931676 29578522

[39] Forgacs L et al. 2020. Comparison of in vivo pathogenicity of four *Candida auris* clades in a neutropenic bloodstream infection murine model. Emerg Microbes Infect. 9(1):1160–1169. 32486923 10.1080/22221751.2020.1771218 PMC7448943

[40] Zuluaga AF et al. 2006. Neutropenia induced in outbred mice by a simplified low-dose cyclophosphamide regimen: characterization and applicability to diverse experimental models of infectious diseases. BMC Infect Dis. 6:55. 16545113 10.1186/1471-2334-6-55 PMC1434751

[41] Schweitzer F et al. 2019. Monocyte and alveolar macrophage skewing is associated with the development of pulmonary arterial hypertension in a primate model of HIV infection. AIDS Res Hum Retrovirus. 35(1):63–74. 10.1089/aid.2018.0132 PMC6343195 30229666

[42] Shipley TW et al. 2010. Persistent pneumocystis colonization leads to the development of chronic obstructive pulmonary disease in a nonhuman primate model of AIDS. J Infect Dis. 202(2):302–312. 20533880 10.1086/653485 PMC2946196

[43] Board KF et al. 2003. Experimental *Pneumocystis carinii* pneumonia in simian immunodeficiency virus-infected rhesus macaques. J Infect Dis. 187(4):576–588. 12599074 10.1086/373997

[44] Wakefield AE et al. 1990. Detection of *Pneumocystis carinii* with DNA amplification. Lancet. 336(8713):451–453. 1974987 10.1016/0140-6736(90)92008-6

[45] Croix DA et al. 2002. Alterations in T lymphocyte profiles of bronchoalveolar lavage fluid from SIV- and *Pneumocystis carinii*-coinfected rhesus macaques. AIDS Res Hum Retrovirus. 18(5):391–401. 10.1089/088922202753519179 11897041

[46] Siminski J , Kidd P, Phillips GD, Collins C, Raghu G. 1991. Reversed helper/suppressor T-lymphocyte ratio in bronchoalveolar lavage fluid from patients with breast cancer and *Pneumocystis carinii* pneumonia. Am Rev Respir Dis. 143(2):437–440. 1846728 10.1164/ajrccm/143.2.437

[47] Cobos Jimenez V , Rabacal W, Rayens E, Norris KA. 2019. Immunization with Pneumocystis recombinant KEX1 induces robust and durable humoral responses in immunocompromised non-human primates. Hum Vaccin Immunother. 15(9):2075–2080. 31348719 10.1080/21645515.2019.1631135 PMC6773377

[48] Woodland DL , Scott I. 2005. T cell memory in the lung airways. Proc Am Thorac Soc. 2(2):126–131. 16113480 10.1513/pats.200501-003AW PMC2713315

[49] Torosantucci A et al. 2005. A novel glyco-conjugate vaccine against fungal pathogens. J Exp Med. 202(5):597–606. 16147975 10.1084/jem.20050749 PMC2212864

[50] Wuthrich M et al. 2015. Calnexin induces expansion of antigen-specific CD4(+) T cells that confer immunity to fungal ascomycetes via conserved epitopes. Cell Host Microbe. 17(4):452–465. 25800545 10.1016/j.chom.2015.02.009 PMC4484745

[51] Aitken SL et al. 2014. Clinical practice patterns in hospitalized patients at risk for invasive candidiasis: role of antifungal stewardship programs in an era of rapid diagnostics. Ann Pharmacother. 48(6):683–690. 24687545 10.1177/1060028014529928

[52] Morris A , Norris KA. 2012. Colonization by *Pneumocystis jirovecii* and its role in disease. Clin Microbiol Rev. 25(2):297–317. 22491773 10.1128/CMR.00013-12 PMC3346301

[53] Morris A et al. 2008. Relationship of pneumocystis antibody response to severity of chronic obstructive pulmonary disease. Clin Infect Dis. 47(7):e64–e68. 18724825 10.1086/591701 PMC2602869

[54] Rayens E et al. 2021. Relationship of Pneumocystis antibody responses to paediatric asthma severity. BMJ Open Respir Res. 8(1):e000842. 10.1136/bmjresp-2020-000842 PMC7993353 33762359

[55] Sallusto F , Lanzavecchia A, Araki K, Ahmed R. 2010. From vaccines to memory and back. Immunity. 33(4):451–463. 21029957 10.1016/j.immuni.2010.10.008 PMC3760154

[56] Esser MT et al. 2003. Memory T cells and vaccines. Vaccine. 21(5-6):419–430. 12531640 10.1016/s0264-410x(02)00407-3

[57] Wang J et al. 2015. Kexin-like endoprotease KexB is required for N-glycan processing, morphogenesis and virulence in *Aspergillus fumigatus*. Fungal Genet Biol. 76:57–69. 25687931 10.1016/j.fgb.2015.02.006 PMC4410318

[58] Gomez FJ , Gomez AM, Deepe GS Jr. 1991. Protective efficacy of a 62-kilodalton antigen, HIS-62, from the cell wall and cell membrane of *Histoplasma capsulatum* yeast cells. Infect Immun. 59(12):4459–4464. 1937804 10.1128/iai.59.12.4459-4464.1991 PMC259063

