# Supplementary material for: Immunogenicity and protective efficacy of a pan-fungal vaccine in preclinical models of aspergillosis, candidiasis, and pneumocystosis
Source: PNAS Nexus. 2022 Nov 4;1(5):pgac248. doi: 10.1093/pnasnexus/pgac248 (PMC9802316; doi:10.1093/pnasnexus/pgac248)
Supplement: pgac248_Supplemental_Files [file pgac248_supplemental_files.zip › PNASNEXUS-PNASNEXUS-2022-00653-T-s01.pptx]

## Slide 1
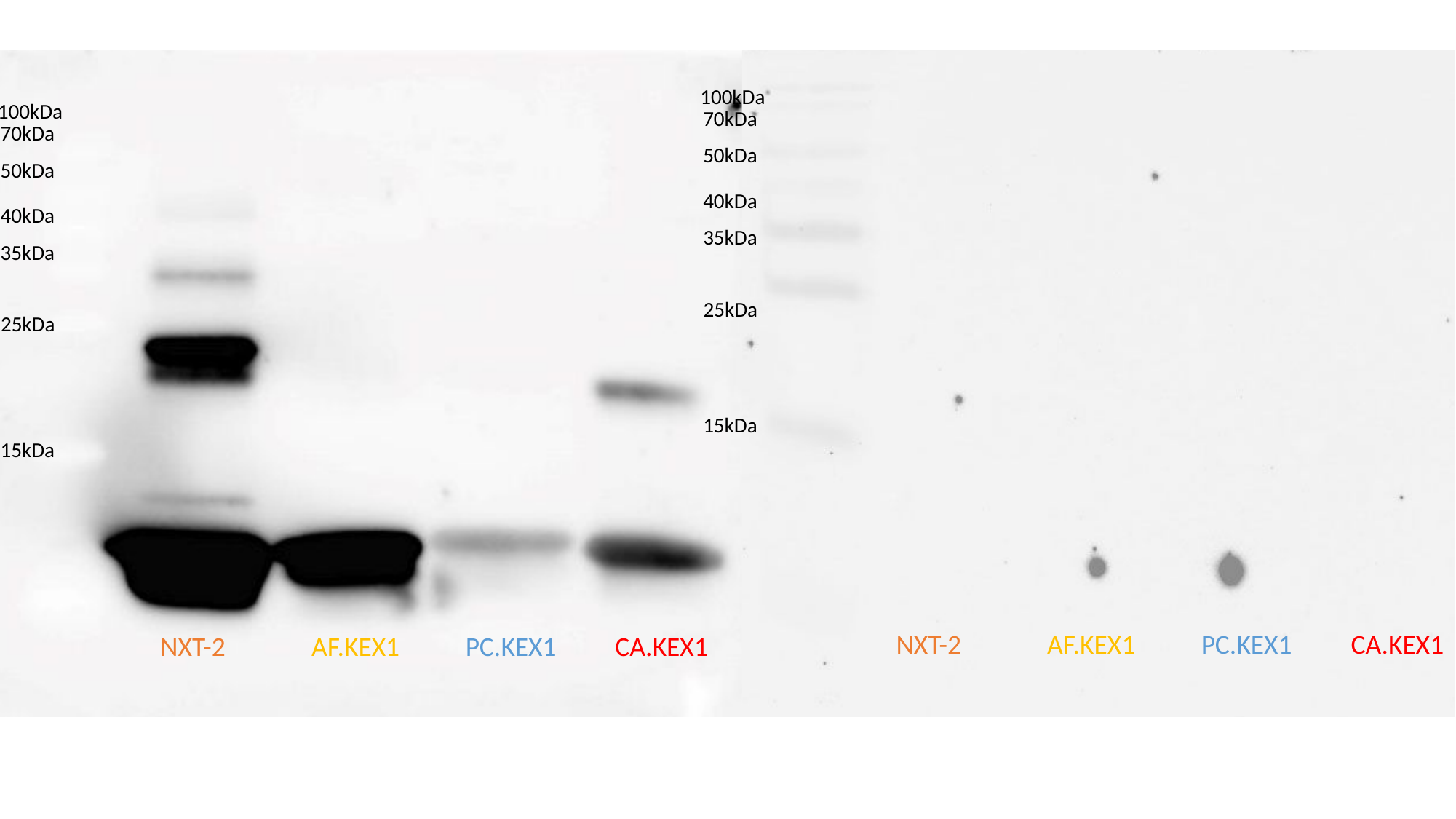

100kDa
100kDa
70kDa
70kDa
50kDa
50kDa
40kDa
40kDa
35kDa
35kDa
25kDa
25kDa
15kDa
15kDa
CA.KEX1
PC.KEX1
AF.KEX1
NXT-2
CA.KEX1
PC.KEX1
AF.KEX1
NXT-2

## Slide 2
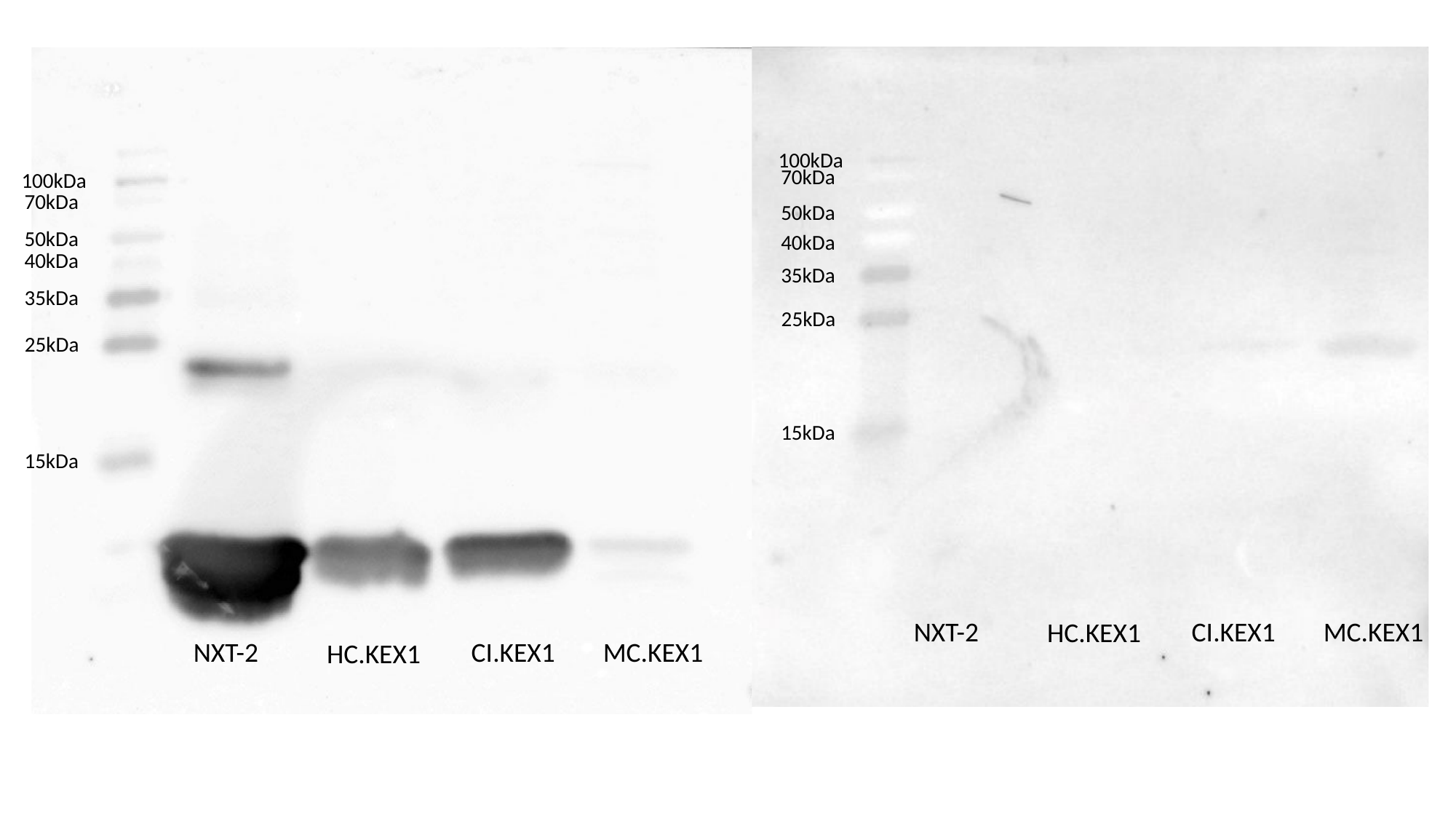

100kDa
70kDa
100kDa
70kDa
50kDa
50kDa
40kDa
40kDa
35kDa
35kDa
25kDa
25kDa
15kDa
15kDa
NXT-2
CI.KEX1
MC.KEX1
HC.KEX1
NXT-2
CI.KEX1
MC.KEX1
HC.KEX1

## Slide 3
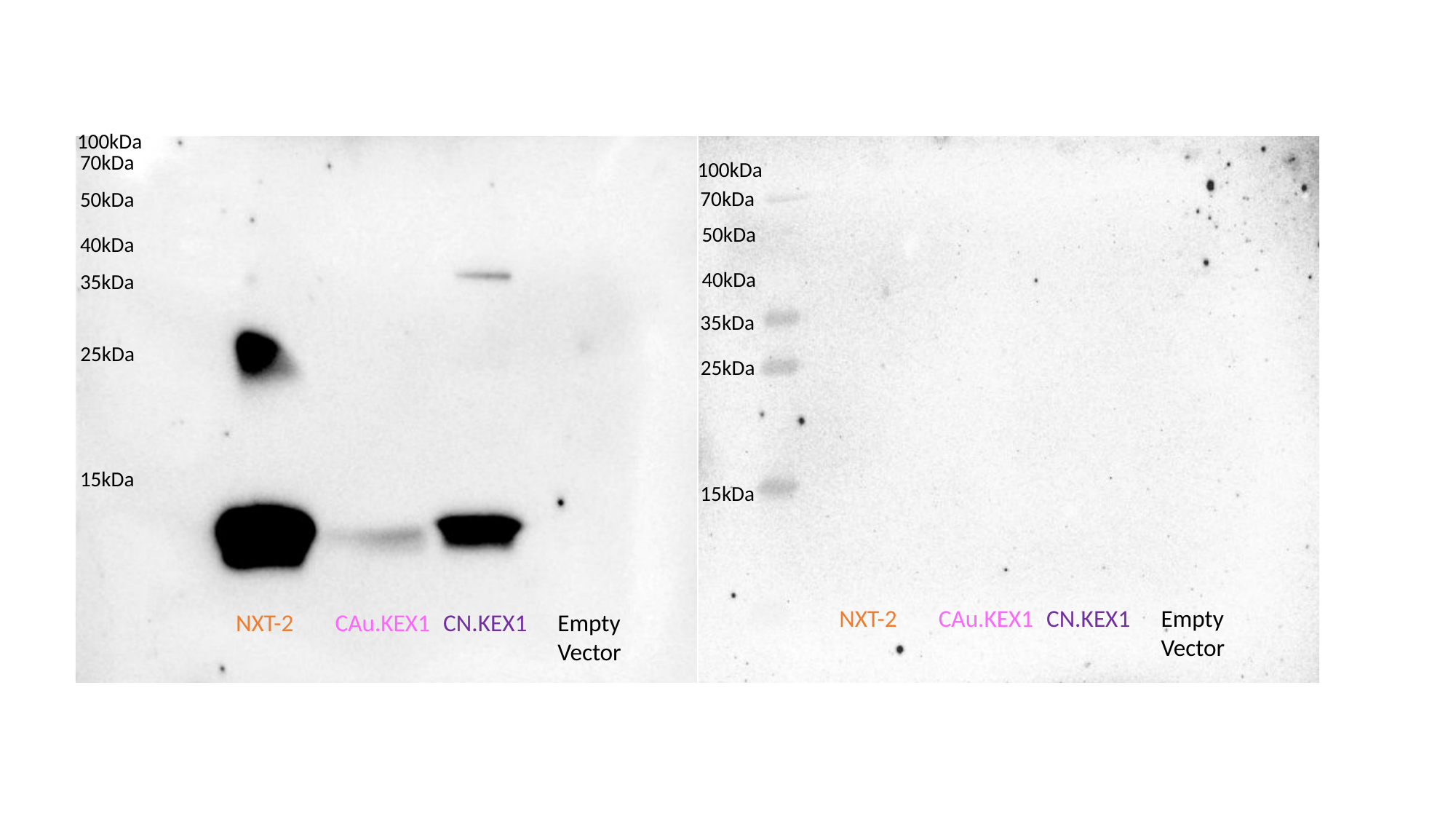

100kDa
70kDa
100kDa
70kDa
50kDa
50kDa
40kDa
40kDa
35kDa
35kDa
25kDa
25kDa
15kDa
15kDa
Empty
Vector
NXT-2
CAu.KEX1
CN.KEX1
Empty
Vector
NXT-2
CAu.KEX1
CN.KEX1
