# Supplementary material for: Immunogenicity and protective efficacy of a pan-fungal vaccine in preclinical models of aspergillosis, candidiasis, and pneumocystosis
Source: PNAS Nexus. 2022 Nov 4;1(5):pgac248. doi: 10.1093/pnasnexus/pgac248 (PMC9802316; doi:10.1093/pnasnexus/pgac248)
Supplement: pgac248_Supplemental_Files [file pgac248_supplemental_files.zip › PNASNEXUS-PNASNEXUS-2022-00653-T-s02.docx]

| **Supplemental Table 1: Vaccine Groups** | | | | | | |
| --- | --- | --- | --- | --- | --- | --- |
| **Species** | **Immunosuppressive Regimen** | **n** | **Sex** | **Vaccine** | **Hyperimmune Plasma Application** | **Challenge** |
| BALB/c  Mice | Tacrolimus and Hydrocortisone | 12 | 5F, 7M | NXT-2+TiterMax | Opsonophagocytic Killing | *Aspergillus fumigatus*  AF293 |
|  |  | 13 | 5F, 7M | PBS+TiterMax |  |  |
|  |  | 13 | 5F, 7M | AF.KEX1+TiterMax* |  |  |
|  | No Immunosuppression | 10 | 5F, 5M | NXT-2+Alhydrogel | Surface Binding | No Challenge |
|  |  | 10 | 5F, 5M | PBS+Alhydrogel |  |  |
| CD-1  Mice | Cyclophosphamide and Cortisone Acetate | 15 | 8F, 7M | NXT-2+TiterMax | Biofilm Inhibition, Opsonophagocytic Killing | *Candida albicans*  SC5314 |
|  |  | 15 | 7F, 8M | PBS+TiterMax |  |  |
|  |  | 15 | 8F, 7M | CA.KEX1+TiterMax |  |  |
| Rhesus macaques | Simian Immunodeficiency Virus | 7 | 7F | NXT-2+Imject Alum | Biofilm Inhibition | Environmental *Pneumocystis* |
|  |  | 8 | 8F | PBS+Imject Alum |  |  |
|  |  | 8 | 8M | No Vaccine |  |  |
|  | No Immunosuppression | 2 | 2F | NXT-2a+Alhydrogel | Western Blot Cross-Reactivity | No Challenge |
| *Previously published work (**33**) | | | | | | |

**Supplemental Figure 1. Cyclophosphamide-Induced Neutropenia.**

Percent depletion of neutrophils in mice 3 days following intraperitoneal injection of 200mg/kg cyclophosphamide. Depletion was determined by relative neutrophil count in blood smears prepared prior to and following cyclophosphamide treatment.

**Supplemental Figure 2. T-helper Skewing in NXT-2 vaccination**. ***(A)*** Peripheral blood lymphocytes collected at 2 weeks after the second immunization were did not significantly differ between Th1 (CXCR3^+^CCR4^−^) and Th2 (CCR4^+^CXCR3^−^) phenotypes. ***(B)*** Th1 (IgG2a) and Th2 (IgG2) antibody levels in pooled plasma from NXT-2+TiterMax-immunized mice.
