# Supplementary figures and images for: Immunogenicity and protective efficacy of a pan-fungal vaccine in preclinical models of aspergillosis, candidiasis, and pneumocystosis
Source: PNAS Nexus. 2022 Nov 4;1(5):pgac248. doi: 10.1093/pnasnexus/pgac248 (PMC9802316; doi:10.1093/pnasnexus/pgac248)

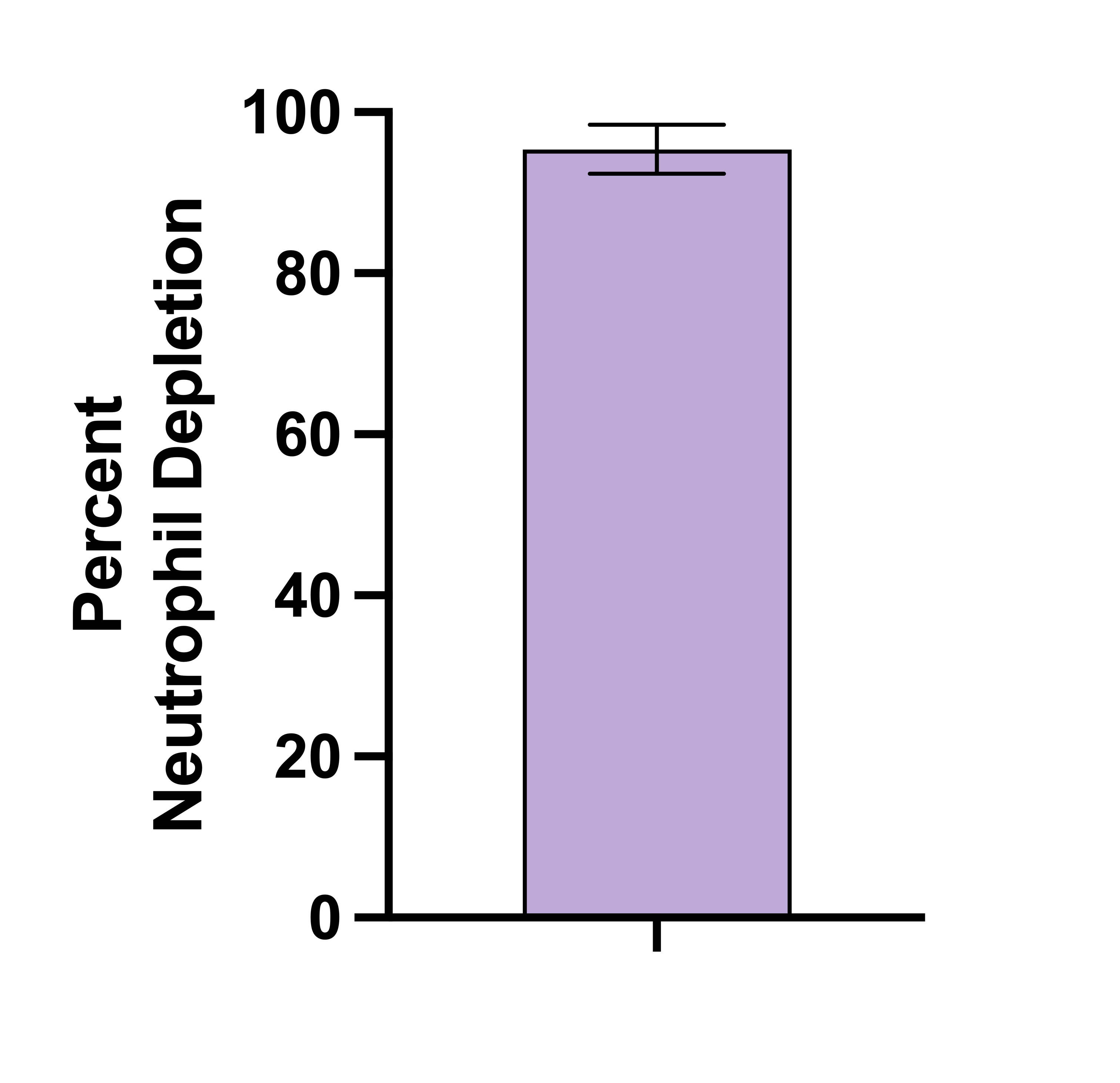

Supplement: pgac248_Supplemental_Files [file pgac248_supplemental_files.zip › PNASNEXUS-PNASNEXUS-2022-00653-T-s03.tif]

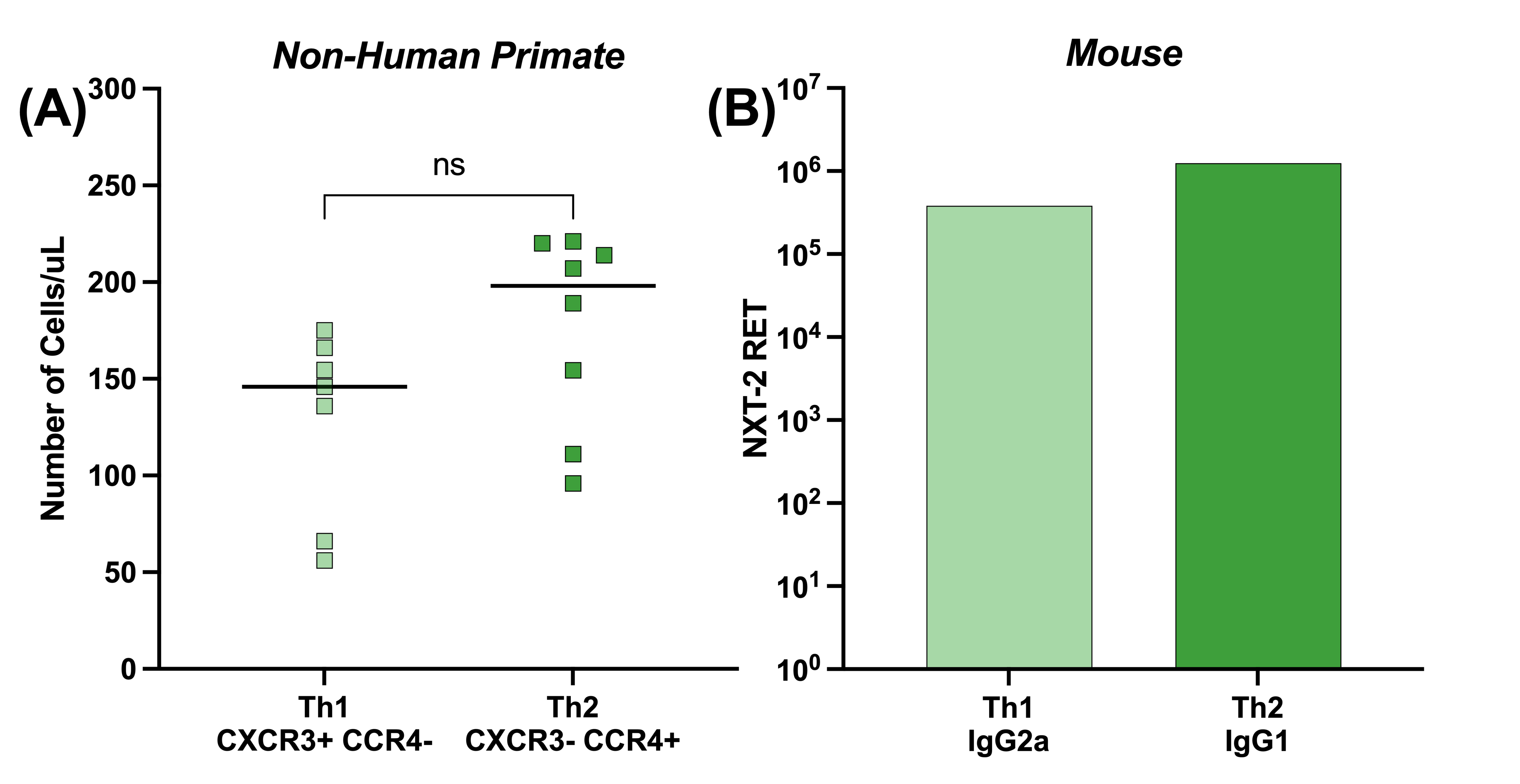

Supplement: pgac248_Supplemental_Files [file pgac248_supplemental_files.zip › PNASNEXUS-PNASNEXUS-2022-00653-T-s04.tif]
